# m^1^A regulator-mediated methylation modification patterns correlated with autophagy to predict the prognosis of hepatocellular carcinoma

**DOI:** 10.1186/s12885-024-12235-4

**Published:** 2024-04-22

**Authors:** Yingmin Wu, Lian Li, Long Wang, Shenjie Zhang, Zhirui zeng, Jieyu Lu, Zhi Wang, Yewei Zhang, Shilong Zhang, Haiyang Li, Tengxiang Chen

**Affiliations:** 1 Department of Physiology, School of Basic Medical Sciences, Guizhou Medical University, 561113 Guiyang, China; 2 Department of Surgery, Affiliated Hospital of Guizhou Medical University, 550004 Guiyang, China; 3 Transformation Engineering Research Center of Chronic Disease Diagnosis and Treatment, Guizhou Medical University, Guiyang, China; 4 Guizhou Provincial Key Laboratory of Pathogenesis and Drug Research on Common Chronic Diseases, Guizhou Medical University, 561113 Guiyang, China; 5 Guizhou Institute of Precision Medicine, Affiliated Hospital of Guizhou Medical University, 550004 Guiyang, China

**Keywords:** N1-methyladenosine (m^1^A), m^1^A regulators, Autophagy, Hepatocellular carcinoma (HCC), Prognosis

## Abstract

**Background:**

N1-methyladenosine (m^1^A), among the most common internal modifications on RNAs, has a crucial role to play in cancer development. The purpose of this study were systematically investigate the modification characteristics of m^1^A in hepatocellular carcinoma (HCC) to unveil its potential as an anticancer target and to develop a model related to m^1^A modification characteristics with biological functions. This model could predict the prognosis for patients with HCC.

**Methods:**

An integrated analysis of the TCGA-LIHC database was performed to explore the gene signatures and clinical relevance of 10 m^1^A regulators. Furthermore, the biological pathways regulated by m^1^A modification patterns were investigated. The risk model was established using the genes that showed differential expression (DEGs) between various m^1^A modification patterns and autophagy clusters. These in vitro experiments were subsequently designed to validate the role of m^1^A in HCC cell growth and autophagy. Immunohistochemistry was employed to assess m^1^A levels and the expression of DEGs from the risk model in HCC tissues and paracancer tissues using tissue microarray.

**Results:**

The risk model, constructed from five DEGs (CDK5R2, TRIM36, DCAF8L, CYP26B, and PAGE1), exhibited significant prognostic value in predicting survival rates among individuals with HCC. Moreover, HCC tissues showed decreased levels of m^1^A compared to paracancer tissues. Furthermore, the low m^1^A level group indicated a poorer clinical outcome for patients with HCC. Additionally, m^1^A modification may positively influence autophagy regulation, thereby inhibiting HCC cells proliferation under nutrient deficiency conditions.

**Conclusions:**

The risk model, comprising m^1^A regulators correlated with autophagy and constructed from five DEGs, could be instrumental in predicting HCC prognosis. The reduced level of m^1^A may represent a potential target for anti-HCC strategies.

**Supplementary Information:**

The online version contains supplementary material available at 10.1186/s12885-024-12235-4.

## Background

Modifications of RNA that are dynamic and reversible can regulate gene expression post-transcriptionally. The chemical modification process has been classified into more than 170 distinct types, including prominent ones such as N6-methyladenosine (m^6^A), 5-methylcytosine (m^5^C), and N1-methyladenosine (m^1^A) [1]. Among these, Post-transcriptional modification m^1^A is extremely common that adorns various RNA types—tRNA, rRNA, mitochondrial RNA, and mRNA—and has been recognized since the 1960s [2]. m^1^A, a modification seen in various gene transcripts in eukaryotic cells ranging from yeast to mammals, is created by introducing a methyl group at the N1 site of adenosine. In the case of humans, the approximate average transcript stoichiometry is believed to be around 20% [3, 4]. The m^1^A’s methyl group is strategically positioned at the Watson-Crick base pairing interface disrupting base pairing. Additionally, positive charge on m^1^A significantly influences structure of local RNA or interactions between proteins and RNA [5].

m^1^A, a post-transcriptional RNA modification that is dynamic and reversible, is regulated by RNA-binding proteins, methyltransferases, and demethylases, which are commonly known as “writers,” “erasers,” and “readers” [6, 7]. The process of m^1^A methylation involves catalysis by methyltransferases such as TRMT6/61A, Trmt61B, and TRMT10C, while demethylation is facilitated by demethylases such as ALKBH1 and ALKBH3. Moreover, m^1^A is recognized by m^1^A-dependent RNA-binding proteins including YTHDF1, YTHDF2, YTHDF3, and YTHDC1 [8]. Recent data suggest dysregulated expression and changes in genetics in m^1^A regulators are associated with tumorigenesis and its progression, causing phenomena such as dysregulated tumor cell death, proliferation, invasion, the tumor microenvironment, and senescence [9–13]. The present study aimed to deeply explore genes variations in m^1^A regulator and construct a model for predicting the prognosis of hepatocellular carcinoma (HCC) based on the gene signatures of these regulators.

Autophagy has garnered significant attention due to its dual role in the maintenance of cancer cells, operating both as an oncogenesis inducer and as a supressor of tumors [14]. The contradictory function of autophagy in tumorigenesis depends on various stages of cancer development and is further influenced by environmental factors, such as nutrient availability, immune system status, pathogenic conditions, and microenvironmental stress [15]. Given this dual nature, attempting to regulate the autophagic process using anticancer drugs could potentially yield fatal outcomes and significantly affect the overall survival (OS) of patients [16]. Therefore, conducting an in-depth analysis of the autophagy status in patients with cancer and understanding the molecular mechanisms regulating autophagy hold promise in predicting prognosis and devising antitumor therapies.

Many lines of evidence indicate that regulators and machineries associated with autophagy undergo epigenetic modulation, leading to alterations in autophagic processes. These alterations subsequently contribute to the development of diseases or affect the efficacy of therapeutic agents [17]. For example, the presence of atypical DNA methylation and demethylation in autophagy-related genes (ATG) is associated with higher tumor grade, stage, lymphatic invasion, and can serve as an indicator of an unfavorable prognosis in individuals diagnosed with cancer [18, 19]. Moreover, the histone lysine methyltransferase G9a can directly interact with the promoters of core ATG, such as LC3B, TP53, and WIPI1 elevating their histone methylation levels, thereby reducing autophagy levels [20]. Regarding RNA modification, the m^6^A reader YTHDF3 recognizes m^6^A modification sites around FOXO3 mRNA’s stop codon. This recognition facilitates FOXO3 translation by recruiting eIF3a and eIF4B, thereby promoting autophagy in cancer cells [21]. Conversely, renal cell carcinoma with clear cells is a disease that is facilitated by the m^6^A eraser FTO-mediated autophagy through the FTO/autophagy/SIK2 axis [22]. In esophageal squamous cell carcinoma, recent studies have also emphasized the role of METTL1 and WDR4, which are part of the tRNA m^7^G methyltransferase complex, in the negative regulation of MTORC1-mediated autophagy [23]. However, the regulatory relationship between autophagy and m^1^A modification in cancer development, along with its role in patients with HCC’s prognosis, remains unknown.

The study utilized genomic data and clinical information from 374 liver HCC (LIHC) samples in The Cancer Genome Atlas (TCGA) database to comprehensively analyze the genetic code variations of m^1^A regulators, m^1^A modification patterns, and their correlation with autophagy characteristics. m^1^A modification patterns with two distinct patterns were identified based on regulators with 10 m^1^A and observed a close association between autophagy characterization and these two distinct m^1^A modification subtypes. Additionally, this study demonstrated the positive role of m^1^A modification in regulating individual tumor autophagy characteristics, including autophagy levels under nutritional deficiency in vitro. Furthermore, our study revealed a decrease in m^1^A modification levels in HCCs, where a low level of m^1^A indicated a poorer clinical outcome for patients with HCC. Finally, a scoring system based on common DEGs between a variety of m^1^A modification patterns and different autophagy clusters was established to assess the prognostic risk in individual patients with HCC.

## Materials and methods

### Data sourcing and pre-processing

Raw data count for RNA-Seq and CNV (Copy number variation), along with associated clinical information from the LIHC cohorts, were obtained from TCGA (https://portal.gdc). The TCGA project comprises 374 cases of HCCs and 50 normal tissue samples. Each normalization technique was implemented using the statistical computing language R, and subsequent data analysis steps were performed using R software version 4.2.1.

### Analysis of the principal components

This study identified 10 genes responsible for m^1^A RNA modification based on prior research. To reduce dimensionality, PCA (Principal component analysis) was applied, and population vectors were projected onto a two-dimensional space for data visualization.

### Kaplan–Meier survival analysis

The Kaplan-Meier techniques were utilized for univariate analysis in order to examine survival, while the log-rank test was employed to assess disparities in survival distributions. These analyses were executed in R utilizing the “survminer”(version 0.4.9) and “survival” (version 3.3.1) packages. The threshold for statistical significance was set at a significance level of *p*<0.05.

### Unsupervised consensus clustering

To categorize patients based on the similarity in expression of m^1^A-related genes, the ConsensusClusterPlus package (version 1.52.1) from Bioconductor was utilized for performing unsupervised hierarchical clustering of the correlation matrix.

### Gene set variation analysis (GSVA)

The purpose of this study is to examine differences in biological processes involving m^1^A clusters with distinct models of modification, Analysis of GSVA enrichment was conducted employing the “GSVA”R package (version 1.38.2). The results suggested a possible link between m^1^A mRNA modification and autophagy.

### Building and verifying the risk model

To examine the gene expression and survival data of patients with Overall Survival (OS), a univariate COX (Cox proportional-hazards model) analysis was conducted. Subsequently, the LASSO (Least absolute shrinkage and selection operator) Cox model was employed to examine genes associated with survival (*p* < 0.05) to discover additional noteworthy survival-related genes by implementing appropriate penalties (lambda). In the end, a predictive risk model was created by using a multivariate Cox regression analysis and applying an information criterion derived from Akaike. Survivability was assessed in the discovery and verification cohorts by evaluating the prognostic significance of survival using Kaplan-Meier and ROC curves (Receiver operating characteristic curve). In Kaplan-Meier survival analyses, a significance threshold of *p* < 0.05 was employed, while for ROC analyses, cutoffs were defined as *p* < 0.05 and AUC ≥ 0.65.

### Cell culture and transfection

Cell lines derived from with humans HCC QGY-7701 (following were QGY) and HepG2 were procured from Procell Inc. (Wuhan, China). These Lines of cells were cultured in Eagle’s medium modified by Dulbecco (Gibco), supplemented with Serum from fetal bovine animals at 10% (FBS; Abwbio, Germany) and a 1% solution of penicillin-streptomycin (Gibco). Using the MycoAlert Mycoplasma Detection Kit, all cells were routinely tested for mycoplasma contamination. Human HCC cell lines QGY and HepG2 were infected with lentivirus carrying pLKD-CMV-EGFP-Puro-U6-shALKBH3 or pLKD-CMV-EGFP-Puro-U6-shNC plasmids (OBiO Technology, Shanghai). Following this, puromycin was used to select cells at a concentration of 1 µg/mL to ensure stable transfection of cells. Western blot analysis was utilized to detect ALKBH3 protein expression. For plasmid transfection, QGY and HepG2 cells were transfected with 2 µg of PPB, PPB-ALKBH3, or m^1^A demethylation catalytically inactive ALKBH3 mutants R122S and L177A plasmid using Lipofectamine 3000 (Invitrogen) per the manufacturer’s instructions.

### Western blot

HCC cells were lysed using western and immunoprecipitation lysis buffers (Beyotime, Shanghai, China). Subsequently, the lysates of the cells were collected by scraping and spun in a centrifuge for 20 min at 12,000 rpm in a pre-cooled centrifuge at 4 °C. Keeping the supernatant on ice and collecting the supernatant.Protein concentration was determined using the Bradford method. The Western blot technique was carried out using a 10% gel made of sodium dodecyl sulfate–polyacrylamide, with a protein load of 10–15 µg.Following the transfer of proteins onto the membrane, 5% skim milk was introduced at room temperature, and then incubated overnight at 4 °C with primary antibodies. Following four rinses with phosphate-buffered saline containing 0.1% Tween 20 (PBST), the membrane (Proteintech, Wuhan, China) was exposed to a secondary antibody at room temperature for a duration of 2 h. Finally, imaging was conducted using an ECL substrate (Boster, Wuhan, China) after three additional washes with PBST. Antibodies used in this study were ALKBH3 (87620, Cell Signaling Technology; 1:1000), P62 (18420-1-AP, Proteintech; 1:500), LC3 (14600-1-AP, Proteintech; 1:500), beclin (11306-1-AP, Proteintech; 1:500), DCAF8L1 (HPA043787, Atla; 1:1000), CDK5R2/p39 (27058-1-AP, Proteintech; 1:500), PAGE1 (bs-6561R, Bioss; 1:500), TRIM36 (bs-9155R, Bioss; 1:500), CYP26B1 (21555-1-AP, Proteintech; 1:500), and alpha-tubulin (66031-1-lg, Proteintech; 1:1000).

### Dot blot assay

Total RNA was extracted from cells using Trizol (Takara) as per the guidelines provided by the manufacturer. Following that, mRNA was purified by using Sigma-Aldrich’s GenElute Messenger RNA (mRNA) Miniprep Kit (Sigma). The same quantities of mRNA that were diluted in a series were placed onto a nylon membrane (Biosharp) and then exposed to ultraviolet light after being denatured at 70 °C for 5 min. To prevent interference, a block was carried out at room temperature for a duration of 1 hour using 5% milk in PBST. Subsequently, the anti-m^1^A antibody (D345-3, MBL; 1:1000) was incubated overnight. Following PBST washed three times for five minutes, a diluted (1:5000) horseradish peroxidase-conjugated antimouse IgG (Proteintech, Wuhan) was incubated at room temperature for an hour with the membranes. A further three times of washing with PBST were performed for five minutes each, followed by signal detection using ECL reagents (Boster, Wuhan).

### Cell proliferation assay

Assessment of proliferation was conducted through CCK8 and colony formation assays. Assay for colony formation, On a 6-well plate, 2000 cells were seeded per well. After cells were incubated at 37 °C for 12 to 20 days, for a minimum of one hour after fixation with 4% paraformaldehyde and staining with 1% crystal violet, photographs were taken of the stained colonies after three rinses. ImageJ software was utilized to count the colonies by examining micrographs of the plates. In the CCK8 assay, an amount of 1.5 ✕10^3^ cells per well was seeded into a 96-well culture plate. In the CCK8 assay, an amount of 1.5✕10^3^ cells per well was seeded into a 96-well culture plate. These cells were then cultured in normal (control) or EBSS. The CCK-8 reagent (APE-BIO) was added to a well, constituting 10% of the total culture medium volume. Cell viability was determined using a spectrophotometer by measuring absorbance at 450 nm.

### Wound healing assay

For assessment of wound healing, after seeding and culture, 90 percent of the cells formed a confluent monolayer. Afterwards, sterile pipettes were used to scratch the cells and FBS-free media was added. Ten randomly chosen fields were examined under a microscope to determine the distances that cells migrate into the scratched area.

### Immunohistochemistry analysis

Six tissue chips (TFHCC-01), each containing 90 HCC tissues and 90 para-carcinoma tissues, were purchased from TUFEIBIO (Shanghai, China). To perform IHC staining, the tissues were dewaxed and dehydrated using a mixture of ethanol and xylene. Then, they were immersed in a sodium citrate solution (0.1 M, pH 6.0; Solarbio, Shanghai, China) to retrieve antigens through high temperatures and high pressures. To prevent nonspecific binding in the tissues, a blocking solution with 0.3% hydrogen peroxide and 5% bovine serum albumin (Servicebio, Wuhan, China) was applied. Following this, primary antibodies were introduced, and the tissues were incubated overnight at 4 °C. After being washed three times with phosphate-buffered saline, the tissues were exposed to secondary antibodies conjugated with HRP (Proteintech, Wuhan, China) for a duration of 2 h. Subsequently, they were stained using HRP-diaminobenzidine. Subsequently, the presence of the antigen-antibody complex was identified in tissues through the utilization of an orthotopic light microscope. The primary antibodies used for targeted proteins were as follows: m^1^A (1:100), DCAF8L1 (1:100), CDK5R2/p39 (1:50), PAGE1 (1:50), TRIM36 (1:50), and CYP26B1 (1:50).

### Statistical analysis

The results of all experiments conducted in triplicate were analyzed using SPSS software (version 19.0) through statistical analysis. Differences between groups were calculated using either a Student’s t-test or a one-way analysis of variance, followed by Tukey’s post-hoc test. Statistically significant were the *p*-values that were less than 0.05.

## Results

### Features of genetic variation of m^1^A regulators in HCCs

**Fig. 1:**
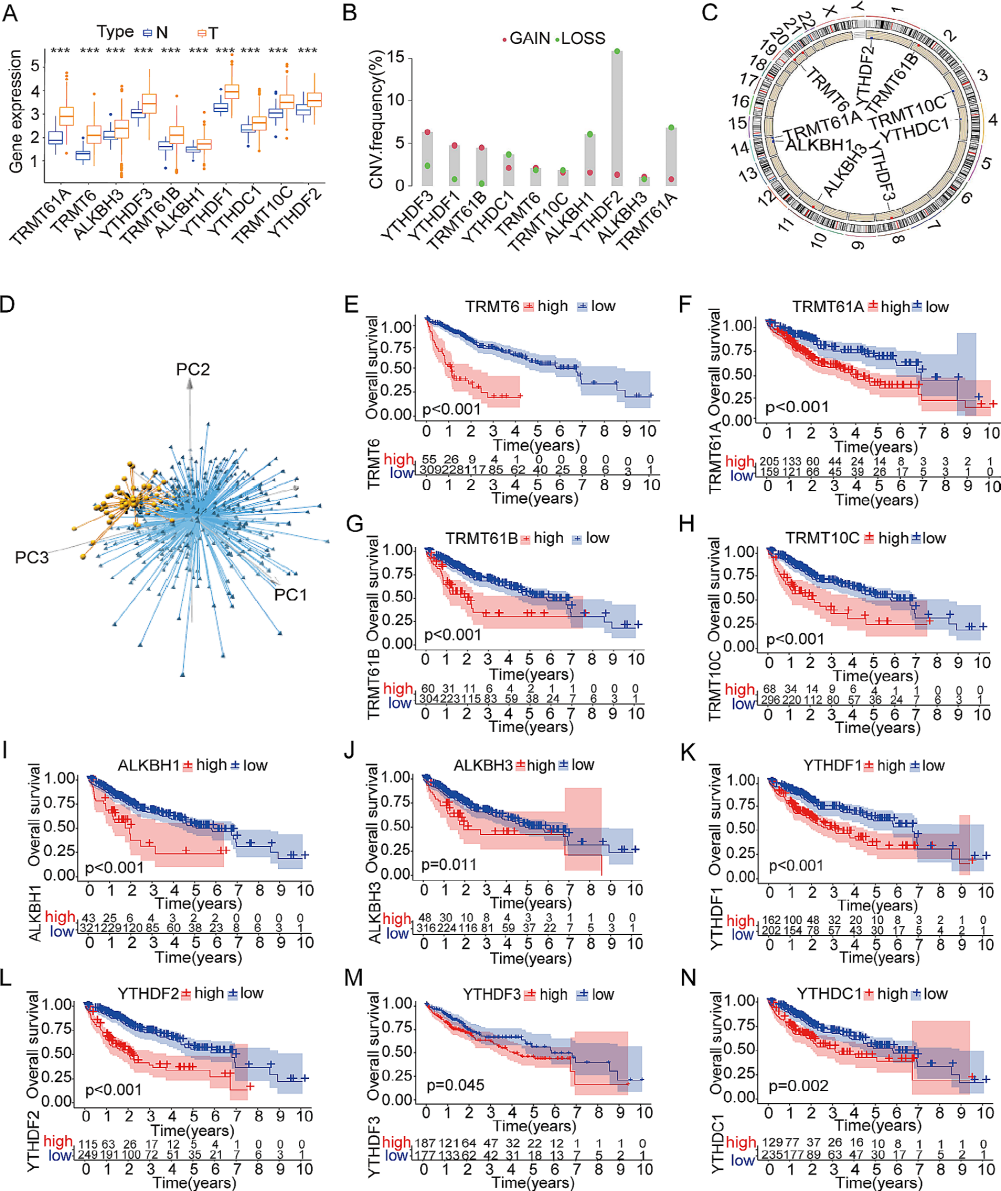
Signatures of genetic variation of m^1^A regulators in hepatocellular carcinoma (HCC) cells. (**A**) Comparative analysis of m^1^A-related genes expressed in tumor and normal liver tissues from The Cancer Genome Atlas-Liver Hepatocellular Carcinoma (TCGA-LIHC); (**B**) Frequency of CNV variation in TCGA-LIHC regulators. Column height represents CNV alteration frequency. Deletion frequency is indicated by green dots, and the amplification frequency is indicated by red dots; (**C**) Location of alterations in copy number variation (CNV) of m^1^A regulators on 23 chromosomes using the TCGA-LIHC cohort; (**D**) Analysis of 10 m^1^A regulator expression profiles in the TCGA-LIHC cohort by principal component analysis (PCA). On the basis of m^1^A regulator expression profiles, we identified two subgroups without intersection, indicating excellent differentiation between tumor samples and normal samples. Tumors are labeled in blue and normal samples in yellow; (**E**–**N**) Overall survival of subgroups of low m^1^A regulator expression and high m^1^A regulator expression, including TRMT6 (**E**), TRMT61A (**F**), TRMT61B (**G**), TRMT10C (**H**), ALKBH1 (**I**), ALKBH3 (**J**), YTHDF1 (**K**), YTHDF2 (**L**), YTHDF3 (**M**), and YTHDC1 (**N**) *p < 0.05, **p < 0.01, ***p < 0.001 compared with the control group; ns, not significant

Drawing on insights from previous studies [1–3], the present investigation analyzed genetic variations in m^1^A regulators, namely TRMT6/61A, TRMT61B, TRMT10C, ALKBH1, ALKBH3, YTHDF1, YTHDF2, YTHDF3, and YTHDC1. To identify the m^1^A regulator landscape in HCC, the gene expression of individual m^1^A regulators was screened in the TCGA database. When comparing the expression between liver tumor tissues and normal liver tissues, significantly elevated levels of the above 10 m^1^A-regulated genes were observed in HCC tissues (Fig. 1A). A prevalent copy number variation (CNV) alteration in m^1^A-regulated genes. In the classification of CNVs, three types were distinguished: amplifications, diploids, and deletions [4]. In particular, TRMT61B, YTHDF1, and YTHDF3 showed significant amplification of copy numbers, while TRMT61A, ALKBH1, YTHDF2, and YTHDC1 had a wide range of CNV deletions (Fig. 1B). The location of CNV alterations in m^1^A-regulated genes on chromosomes is illustrated in Fig. 1C, revealing that gene expression levels can be affected by CNV alterations through compensation effects of dose [24, 25]. The expression patterns of these 10 m^1^A-regulated genes made it feasible distinctly separate HCC samples from normal samples (Fig. 1D). The analyses reveal the significant diversity in the genetic and expression changes of the m^1^A regulators in both normal and HCC tissues, emphasizing the crucial importance of imbalanced gene expression controlled by m^1^A in the development and advancement of HCC.

To further elucidate the influence of m^1^A-regulated gene expression levels on the prognosis, HCC patients were categorized into groups with high- and low-expression. Kaplan–Meier curve analysis demonstrated that higher expression levels of the aforementioned 10 regulators, including TRMT6, TRMT61A, TRMT61B, TRMT10C, ALKBH1, ALKBH3, YTHDF1, YTHDF2, YTHDF3, and YTHDC1, correlated with poorer clinical outcomes for patients with HCC (*p* < 0.05, Fig. 1E–N). Based on these findings suggest a close association between the expression of m^1^A regulators and patients’ prognoses with HCC, emphasizing the significant role played by dynamic regulation of m^1^A modification in the onset and progression of HCC.

### Modification patterns of m^1^A mediated by 10 regulators

**Fig. 2:**
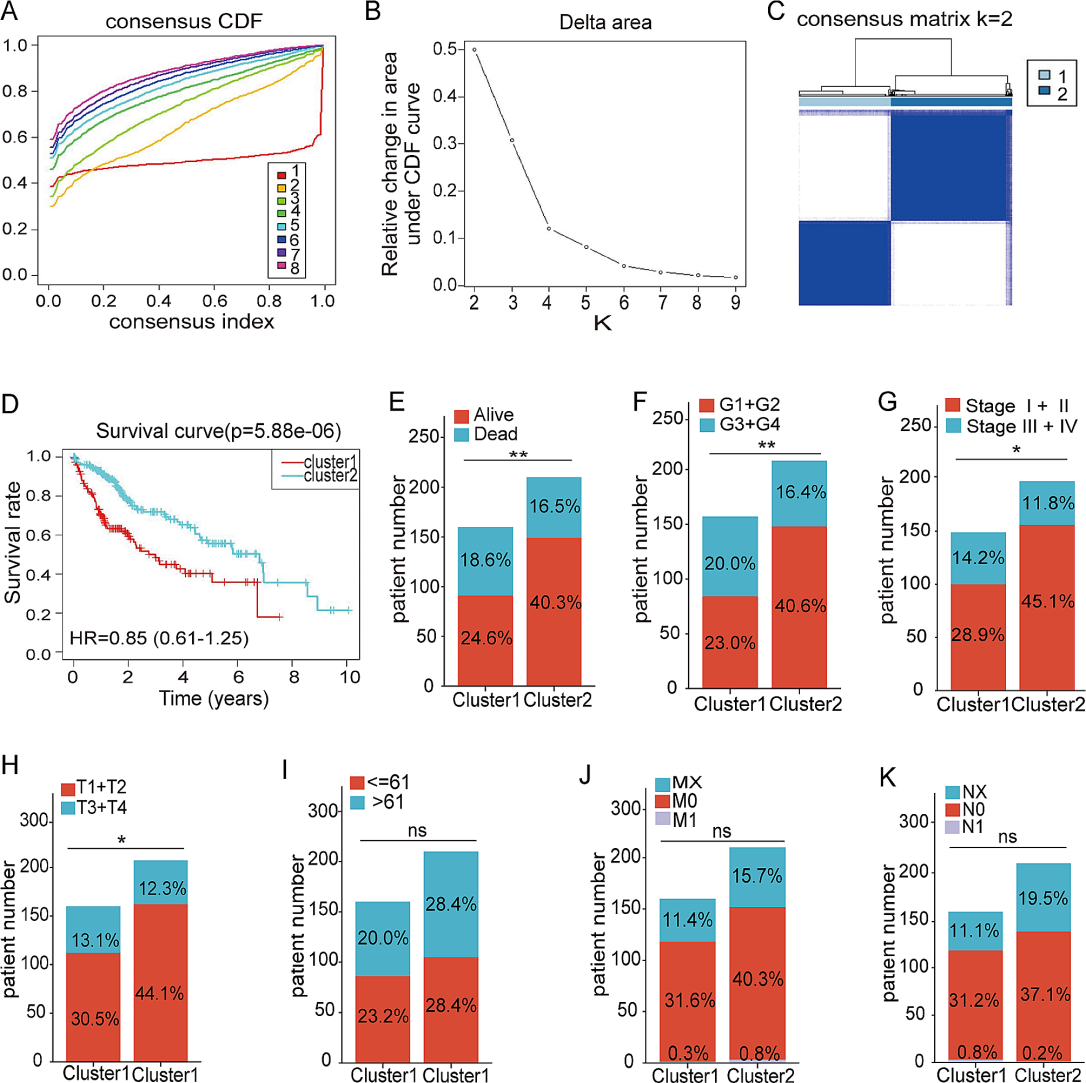
A modification pattern for m^1^A is constructed based on the expression of ten regulators. (**A**) Cumulative distribution function (CDF) curve in The Cancer Genome Atlas–Liver Hepatocellular Carcinoma (TCGA-LIHC) cohort; (**B**) Delta area curve of CDF in TCGA-LIHC cohort; (**C**) Clustering heatmap when consensus k = 2; (**D**) In the TCGA-LIHC cohort, Kaplan-Meier curves show a prognostic relationship between two different m^1^A patterns; (**E**–**K**) Survival status (**E**), grade (**F**), stage (**G**), T stage (**H**), age (**I**), M stage (**J**), and N stage (**K**) of the two different m^1^A patterns of cluster 1 and cluster 2 *p < 0.05, **p < 0.01, ***p < 0.001 compared with the control group; ns, not significant

In order to further investigate the influence of different m^1^A modification patterns on the clinical results, patients were classified according to the expression traits of the regulators for 10 m^1^A using the R software ConsensusClusterPlus and limma. The results yielded a clustering between k = 1 to 8 (Fig. 2A). After configuring clusterAlg as ‘pam’ and distance as ‘euclidean’, and analyzing the cumulative distribution function (CDF) and the CDF delta derived from the area under the curve (AUC), it was determined that the optimal quantity of clusters is 2. This conclusion is supported by the consistent clustering outcome observed in cluster number 2 (Fig. 2B). To obtain two separate m^1^A clusters, cluster 1 (*n* = 189) and cluster 2 (*n* = 185; Fig. 2C), k = 2 was chosen. Analyzing the prognosis for the two m^1^A modification patterns revealed that cluster 2 tended to exhibit a significant advantage in survival (Fig. 2D).

In order to further explore the connection between m^1^A modification patterns and the clinical traits and biological behaviors of individuals with HCC, a comparative analysis of distinct patterns concerning various clinical characteristics was conducted. The findings revealed significant differences in survival status, grade, stage, and T stage between the two different m^1^A modification patterns (Fig. 2E–H, all *p* < 0.05). However, with regard to age, M stage, or N stage, no discernible differences were observed in the m^1^A modification patterns (Fig. 2I–K, all *p* > 0.05). These data suggest a potential strong correlation between m^1^A modification patterns and the prognosis as well as progression of patients with HCC.

### High m^1^A modification inhibits the proliferation and migration of HCC cells

**Fig. 3:**
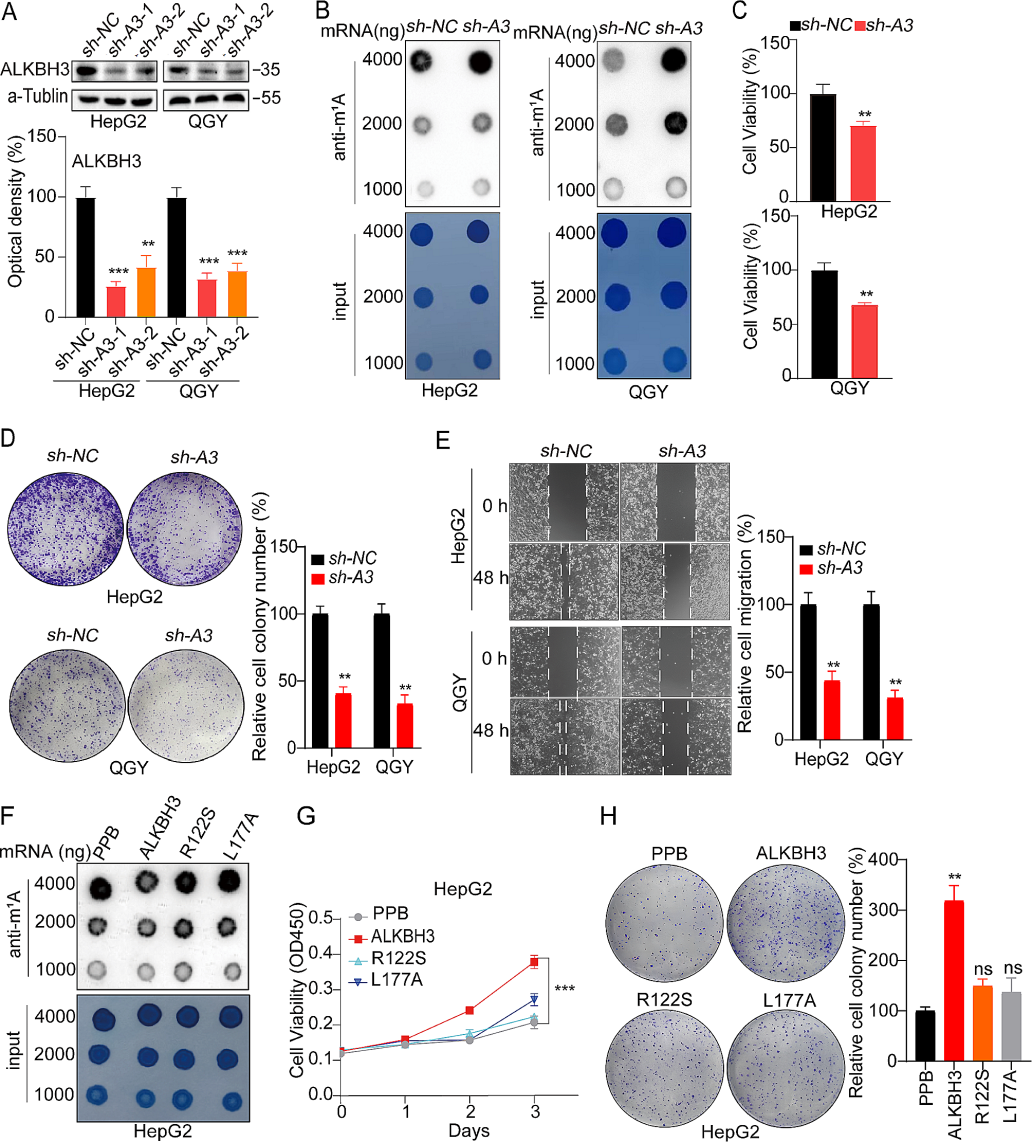
High m^1^A modification inhibits HCC cell proliferation and migration. (**A**) ALKBH3 expression detected by western blotting (up) and quantitatively analyzed (down); (**B**) Levels of mRNA m^1^A modification in sh-ALKBH3 HepG2, sh-ALKBH3 QGY, and their corresponding control cells assessed through dot blot analysis; (**C**) Relative cell viability of sh-ALKBH3 HepG2, sh-ALKBH3 QGY, and their corresponding control cells; (**D** and **E**) Results of colony formation (**D**) and migration (**E**) assays to detect the effects of m^1^A modification on HepG2 and QGY cells; (**F** and **G**) Levels of mRNA m^1^A modification (**F**) and relative cell viability (**G**) of HepG2 cells transfected with vector control (PPB), ALKBH3, ALKBH3-R122S, or ALKBH3-L177A constructs for 24 h (**H**) Results of colony formation of HepG2 cells transfected with vector control (PPB), ALKBH3, ALKBH3-R122S, or ALKBH3-L177A. Data are presented as mean ± standard deviation from three independent experiments. *p < 0.05, **p < 0.01, ***p < 0.001 compared with the control group

m^1^A regulators play a crucial role in dynamically regulating the level of m^1^A modifications [26]. In order to comprehend the significance of various levels of m^1^A modification in HCC, a distinct pattern marked by elevated m^1^A modification levels was established. This was achieved by utilizing two ALKBH3 short hairpin RNA (shALKBH3) constructs, specifically sh-A3-1 and sh-A3-2, to generate stable knockdown HepG2 cells and QGY cells, following the methodology outlined in our prior study (Fig. 3A) [4], as ALKBH3 is identified as the only eraser for mRNA m^1^A [5–6]. And the knockdown efficiency of sh-A3-1 surpassed that of sh-A3-2,consequently, sh-A3-1 was selected for subsequent experiments (hereinafter referred to as sh-A3). Dot blot analysis indicated significantly higher levels of m^1^A in mRNA in HepG2 and QGY sh-A3 cells than in control cells (Fig. 3B). Knockdown of ALKBH3 led to the suppression of growth (Fig. 3C), colonization (Fig. 3D), and migration (Fig. 3E) in HepG2 and QGY cells. In contrast, the introduction of ALKBH3 into HepG2 cells (which produced a model with reduced m^1^A modification, as mentioned in a previous report) [27], along with ALKBH3 mutants R122S and L177A. The levels of m^1^A in mRNA were checked by dot blot analysis and indicated significantly lower levels of m^1^A in mRNA in HepG2 ALKBH3 overexpressed cells than in control cells, but not in the R122S or L177A mutants overexpressed cells (Fig. 3F). CCK8 and colony formation assays showed that the overexpression of ALKBH3 WT constructs led to enhanced cell proliferation (Fig. 3G) and colonization (Fig. 3H), whereas the R122S or L177A mutants did not have the same effect. These findings imply that a high m^1^A modification level prevents HCC cells from proliferating and migrating.

### Identifying autophagic clusters associated with m^1^A modification patterns

**Fig. 4:**
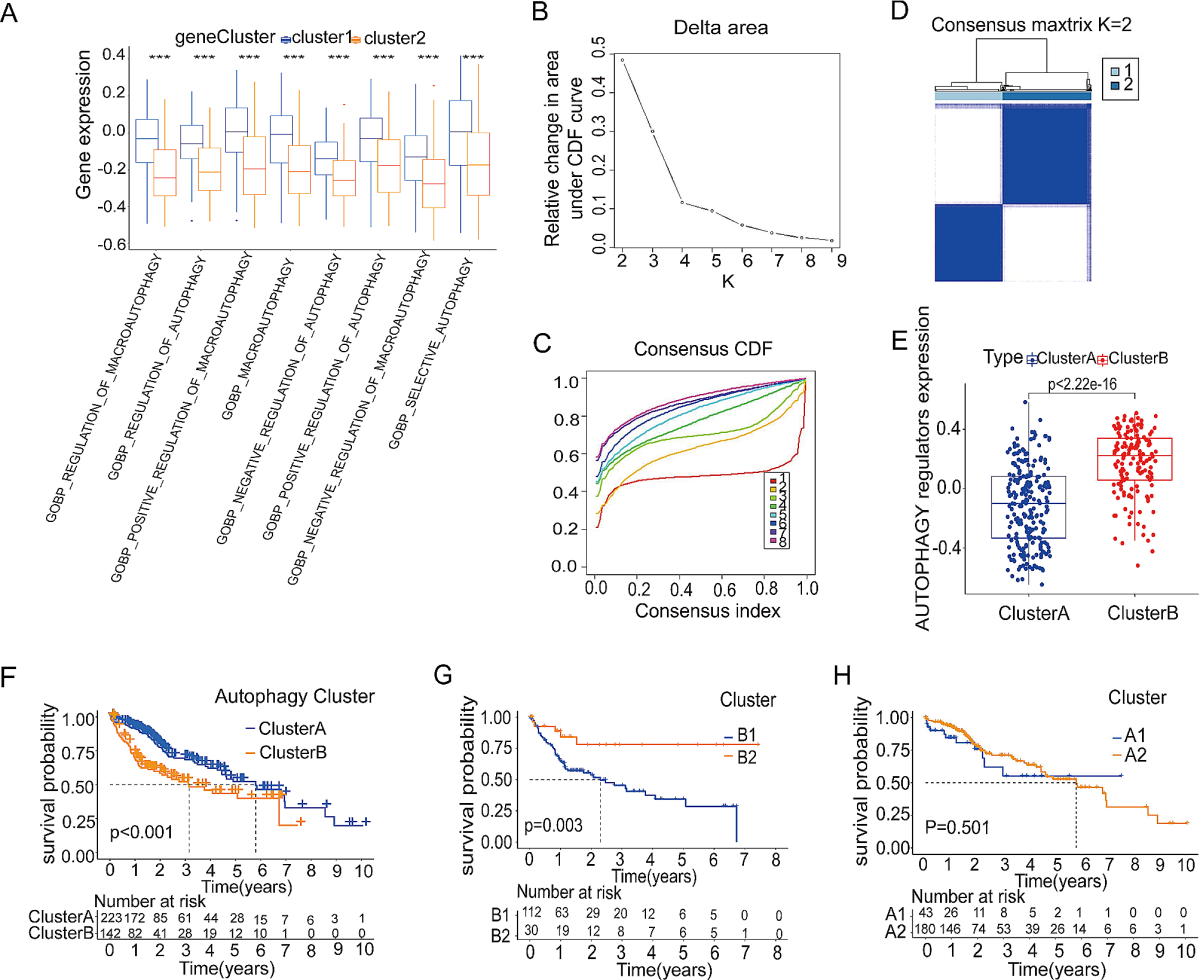
Identification of autophagy clusters related to m^1^A modification patterns. (**A**) Gene set variation enrichment analysis under different m^1^A clusters; (**B**) The delta area curve for the cumulative distribution function (CDF) in the Liver Hepatocellular Carcinoma Cohort of The Cancer Genome Atlas (TCGA-LIHC); (**C**) Curves for the CDF in the TCGA-LIHC cohort; (**D**) Heatmap of clustering when consensus k = 2; (**E**) Based on the TCGA-LIHC cohort, the Kaplan-Meier curve shows a prognostic relationship between cluster A and cluster B; (**F**) Single-sample gene set enrichment analysis of autophagy under different autophagy clusters; (**G** and **H**) Kaplan–Meier curve of the prognostic relationship between B1 and B2 (**G**) or A1 and A2 (**H**). *p < 0.05, **p < 0.01, ***p < 0.001 compared with the control group; *p* > 0.05, ns, not significant

In order to delve deeper into the biological behaviors linked to the two different m^1^A modification patterns (Fig. 2C), we conducted gene set variation analysis (GSVA) enrichment analysis. Figure 4A demonstrates a significant enrichment of m^1^A modification patterns in the autophagy pathway. Subsequently, by analyzing the expression of the 232 ATGs (Table S1) using the ConsensusClusterPlus R package, clustering between k = 1 to 8 was observed (Fig. 4B-C). Employing the same methodology as that used for m^1^A modification patterns to observe the CDF delta, the optimal number of clusters was found to be k = 2 (Fig. 4D). Therefore, two different autophagic clusters were found in 374 HCC patients, one named cluster A (*n* = 225) and the other named cluster B (*n* = 149). Further analysis of the positive regulators among 323 ATGs in the two different autophagy subtypes by ssGSEA on sangerbox 3.0 (http://vip.sangerbox.com) revealed that patients with HCC in cluster B tended to exhibit a higher autophagy phenotype (Fig. 4E). Moreover, compared with autophagy subtypes of cluster A, patients in cluster B experienced poorer prognostic outcomes (Fig. 4F), indicating significant differences in the autophagy status between cluster A and cluster B.

Among patients with HCC in autophagy subtypes of cluster A (defined as the low autophagy status group), 118 patients also belonged to m^1^A modification subtypes cluster 1 (A1) and 31 patients to cluster 2 (A2). Similarly, among patients with HCC in autophagy subtypes of cluster B (defined as the high autophagy status group), 44 patients also belonged to m^1^A modification subtypes cluster 1 (B1) and 181 patients to cluster 2 (B2). To further explore whether m^1^A modification affects the survival and prognosis of patients with HCC through autophagy, groups A1 and A2 or B1 and B2 were selected for survival and prognostic analyses. Through Kaplan Meier curve analysis, the 5-year survival rate of B1 patients was significantly better than that of B2 patients (72.5% vs. 31.3%), and significant survival benefits were demonstrated Fig. 4G. Conversely, the survival rate between patients in A1 and A2 showed no significant difference (Fig. 4H), indicating that different m^1^A modification patterns hold a prognostic value for patients with HCC only in cases of high autophagy status.

### Exploration of mRNA m^1^A modification was associated with autophagy in HCC cells

**Fig. 5:**
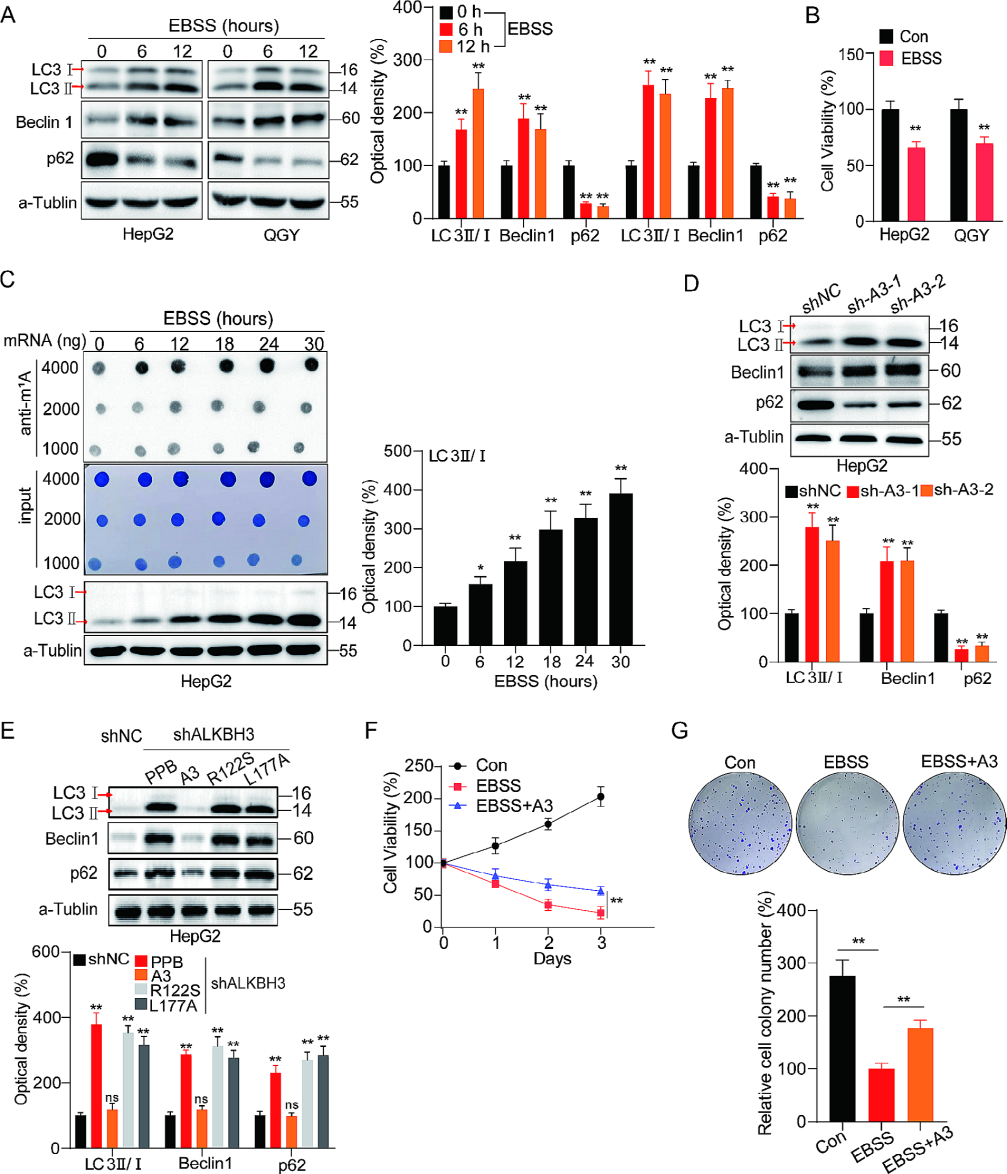
Exploration of mRNA m^1^A modification was associated with autophagy in hepatocellular carcinoma (HCC) cells. (**A**) Expression of LC3I, LC3II, Beclin 1, and p62 in Earle’s balanced salt solution (EBSS)–treated HepG2 or QGY cells for 6, 12 h and their corresponding control cells assessed using western blot (left) and quantitatively analyzed (right); (**B**) Cell proliferation assays to detect the effects of EBSS on HCC cells; (**C**) Levels of mRNA m^1^A modification in EBSS-treated HepG2 cells for 0, 6, 12, 18, 24, and 30 h assessed through dot blot assay. Expression of LC3I and LC3II in the above groups assessed through western blotting and quantitatively analyzed (right); (**D**) Expression of LC3I, LC3II, Beclin 1, and p62 in sh-ALKBH3 HepG2 cells and their corresponding control cells assessed through western blot (up) and quantitatively analyzed (down); (**E**) shNC and shALKBH3 HepG2 cells were transfected with vector control, ALKBH3, ALKBH3-R122S, or ALKBH3-L177A constructs for 48 h, the expression of LC3I, LC3II, Beclin 1, and p62 were checked (up) and quantitatively analyzed (down); (**F** and **G**) Relative fold of the proliferation (**F**) and colonization (**G**) of HepG2 cells treated with EBSS and the ALKBH3 overexpression group and the control group. Data are presented as mean ± standard deviation from three independent experiments. *p < 0.05, **p < 0.01, ***p < 0.001 compared with the control group

To explore the association between m^1^A modification and autophagy in HCC by culturing HepG2 cells and QGY cells in Earle’s balanced salt solution (EBSS) for 6 and 12 h to generate an autophagy model of HCC cells (Fig. 5A) [28]. Notably, cell proliferation significantly decreased upon induced autophagy (Fig. 5B). By analyzing dot blot and protein blot, it is not difficult to observe that the expression of LC3, a classical autophagy marker, increased with the levels of mRNA m^1^A modification in HepG2 cells (Fig. 5C). Moreover, in HepG2 cells exhibiting high mRNA m^1^A modification (HepG2 sh-A3), autophagy was activated (Fig. 5D). Overexpression of ALKBH3 WT constructs, while not R122S or L177A mutant, reversed the activation effect of autophagy in shALKBH3 HepG2 cells (Fig. 5E). Additionally, under autophagy conditions, overexpression of ALKBH3 (here as A3) significantly attenuated the inhibition of cell proliferation (Fig. 5F) and colonization (Fig. 5G) induced by EBSS treatment in HepG2 cells. These findings collectively suggest that mRNA m^1^A modification may act as an autophagy regulator, thereby strongly inhibiting HCC cell proliferation by activating autophagy.

### The construction of the prognostic model is based on the common DEGs between different m^1^A modification patterns and different autophagic clusters

**Fig. 6:**
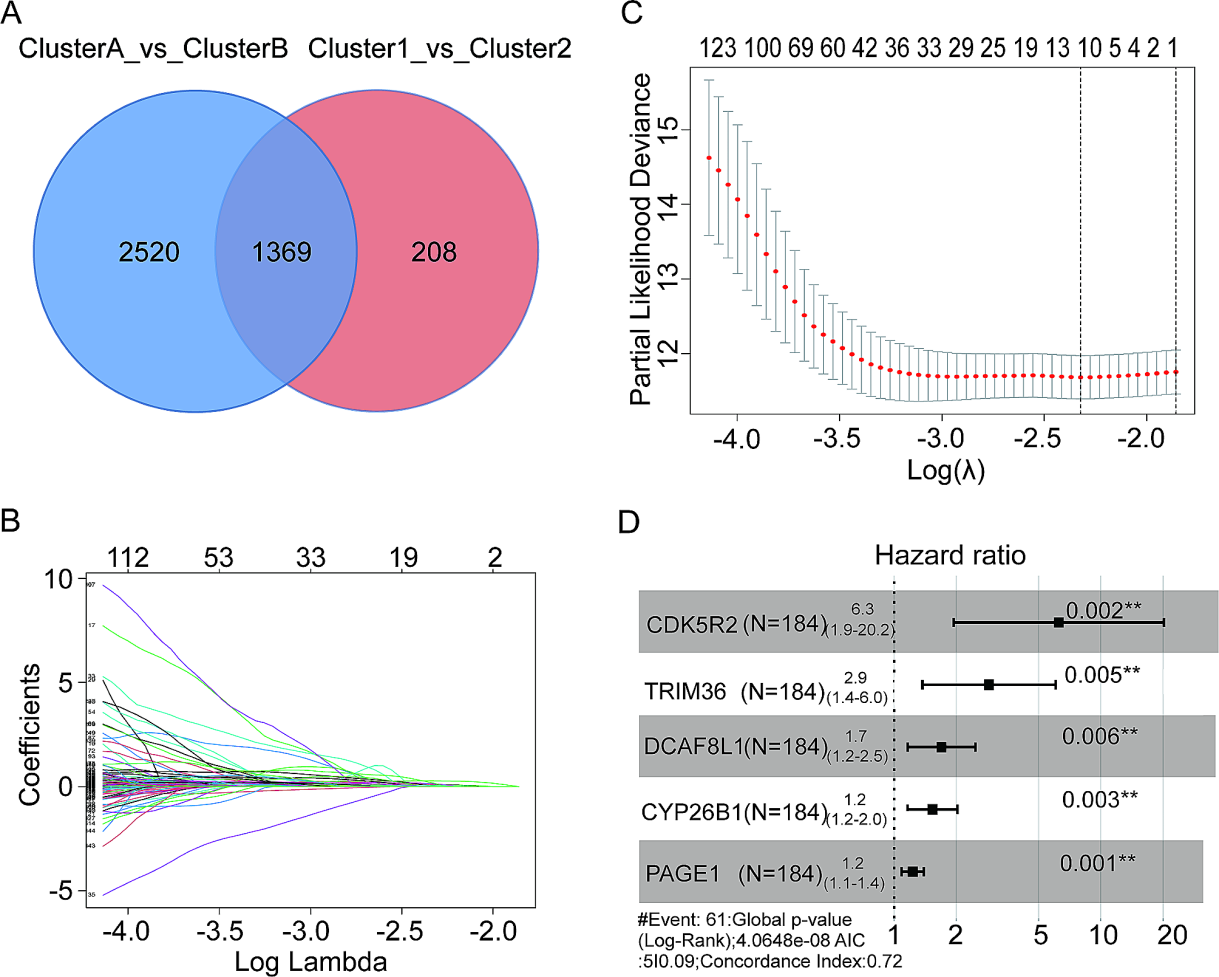
Construction of a prognostic model. (**A**) Common DEGs between various m^1^A modification patterns and autophagy clusters; (**B**) Trajectory change for each independent variable; (**C**) Confidence interval under each lambda; (**D**) Hazard ratio and *p* value of genes (including CDK5R2, TRIM36, DCAF8L1, CYP26B1, and PAGE1) under multivariate Cox regression analysis

In order to advance, a predictive risk model was created to discover potential predictive biomarkers from genes that are expressed differently (DEGs) and are associated with specific m^1^A modification patterns and autophagy clusters. Initially, we examined the shared DEGs in the entire patient population with HCC using various m^1^A modification patterns and autophagic clusters from the TCGA database. The analysis of the intersection showed the existence of 1369 DEGs (Fig. 6A). Afterwards, specimens from 374 individuals diagnosed with HCC sourced from the TCGA-LIHC dataset were randomly split into a training group (*n* = 186) and a validation group (*n* = 188; patients’IDs can be found in Table S2). Within the training set, an analysis was conducted using univariate Cox regression on the aforementioned 1369 DEGs alongside survival data, identifying 534 DEGs associated with a reduction in OS for patients with HCC. Furthermore, in order to improve the accuracy of these 534 DEGs, we utilized LASSO Cox regression analysis to obtain the trajectory of each individual factor, as illustrated in Fig. 6B. As the lambda value gradually increased, the number of coefficients for independent variables approaching zero also increased, as depicted in Fig. 6C. The model reached its optimum at lambda = − 2.318182; therefore, this lambda value was chosen, resulting in the selection of 11 DEGs for further analysis. In the TCGA training group, a prognostic pattern for individuals with HCC was created using CDK5R2, TRIM36, DCAF8L, CYP26B, and PAGE1, which were identified as significant risk predictors through stepwise multivariate Cox regression analysis of these 11 DEGs (Fig. 6D). The risk score calculation involved (1.834 × CDK5R2 expression) + (1.057 × TRIM36 expression) + (0.528 × DCAF8L1 expression) + (0.427 × CYP26B expression) + (0.206 × PAGE1 expression).

### High prognostic value of the prognostic risk model in the training cohort

**Fig. 7:**
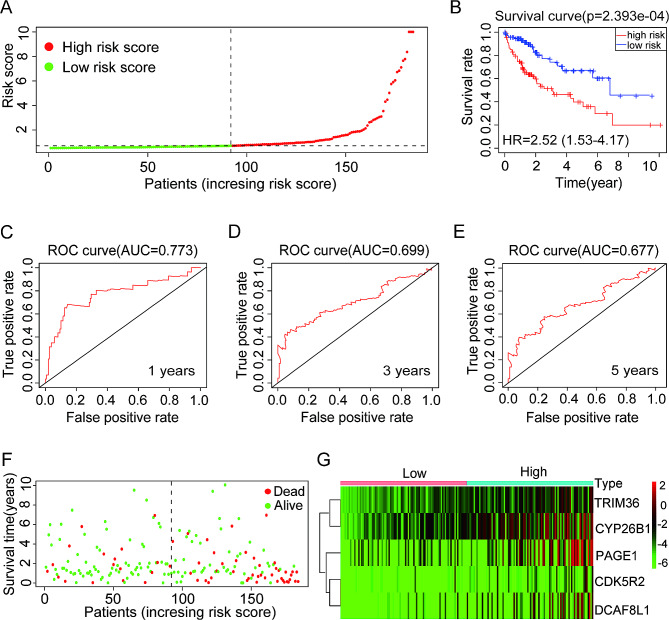
Prognostic risk model showing a high prognostic value in The Cancer Genome Atlas (TCGA) training cohort. (**A**) TCGA training cohort samples of hepatocellular carcinoma (HCC) divided into low- and high-risk groups; (**B**) The overall survival rate of low-risk and high-risk patients with HCC in the TCGA training cohort was analyzed using Kaplan-Meier plots; (**C**–**E**) An indication of the diagnostic value of the risk model for 1-year (**C**), 3-year (**D**), and 5-year (**E**) survival rates in the TCGA training cohort for patients with HCC; (**F**) Survival time and survival status of each patient with HCC in the TCGA training cohort; (**G**) In the TCGA training cohort, the expression of TRIM36, CYP26B1, PAGE1, CDK5R2, and DCAF8L1 was examined in tissues from patients with HCC with high- and low-risk scores

Utilizing the prognostic risk model established in the TCGA training cohort and categorizing patients based on medium-risk scores, patients with HCC were divided into high-risk and low-risk groups (Fig. 7A). Kaplan–Meier curve analysis illustrated that the high-risk group exhibited a lower OS rate (Fig. 7B). Subsequently, the receiver operating characteristic (ROC) curves were analyzed to predict the AUCs values for 1-year, 3-year, and 5-year survival rates of patients with HCC in the TCGA training cohort, the AUC values were 0.773, 0.699 and 0.677, respectively (Fig. 7C–E). Furthermore, individuals diagnosed with HCC and belonging to the high-risk category experience a considerably elevated fatality rate (Fig. 7F). The analysis of the heatmap showed that TRIM36, CYP26B1, PAGE1, CDK5R2, and DCAF8L1 had elevated expression levels in the high-risk score group among the HCC tissues of the TCGA training cohort (Fig. 7G), confirming their significant prognostic value in the TCGA training cohort.

### Verification of the prognostic risk model in the test cohort

**Fig. 8:**
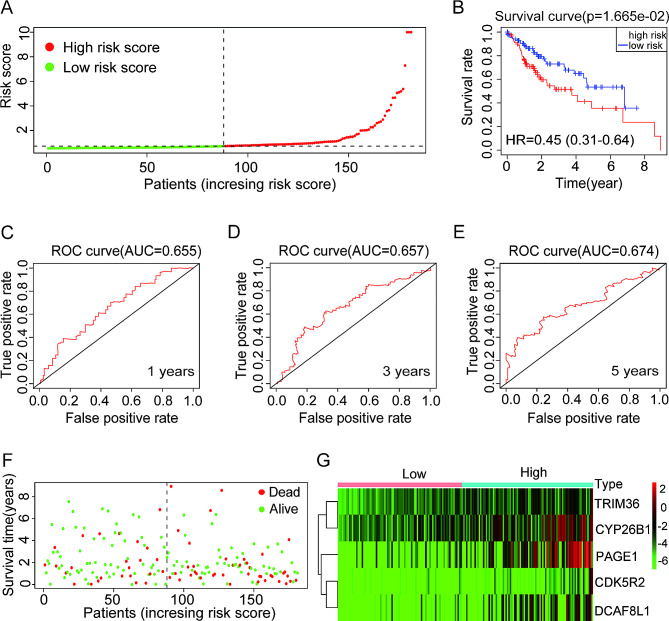
Construction and examination of the risk model in the TCGA test cohort. (**A**) HCC samples from the TCGA test cohort were classified as low-risk or high-risk; (**B**) In the TCGA test cohort, a Kaplan-Meier plot was used to compare TCGA high-risk and low-risk patients with HCC. (**C**–**E**) The risk model provides a diagnostic value for predicting survival rates at 1-year, 3-year, and 5-year follow-up in TCGA cohorts with HCC (**C**, **D**, and **E**); (**F**) A comparison of survival time and survival status of all patients with HCC in the TCGA test cohort; (**G**) Expression levels of TRIM36, CYP26B1, PAGE1, CDK5R2, and DCAF8L1 in HCC tissues collected from low-risk and high-risk patients in the TCGA test cohort

To assess the suitability of prognostic risk models in patients with HCC exhibiting diverse clinical characteristics, a total of 188 HCC patient samples from the TCGA database were utilized as the experimental cohort. Based on the prognostic risk model developed in the TCGA training dataset and the criteria for determining a moderate risk score, HCC tissues with elevated risk scores in the experimental dataset can be categorized into high-risk scoring groups, whereas those with low risk scores can be categorized into low-risk scoring groups (Fig. 8A). Significantly, individuals diagnosed with HCC in the high-risk category demonstrated a decreased overall survival rate compared to those in the low-risk category (Fig. 8B). In the TCGA test database, the AUC values at 1, 3, and 5 years were 0.655, 0.657, and 0.674, respectively (Fig. 8C–E). Corresponding to the training cohort results, patients with HCC who have high-risk scores in the test cohort experienced higher mortality rates (Fig. 8F). Similarly, elevated expression of TRIM36, CYP26B1, PAGE1, CDK5R2, and DCAF8L1 was observed in HCC tissues displaying a high-risk score in the test cohort (Fig. 8G). Overall, the prognostic risk model based on these five genes demonstrated robust predictive efficiency and significant prognostic value for patients with HCC.

### Analysis of the expression characteristics of the five DEGs in HCC tissues

**Fig. 9:**
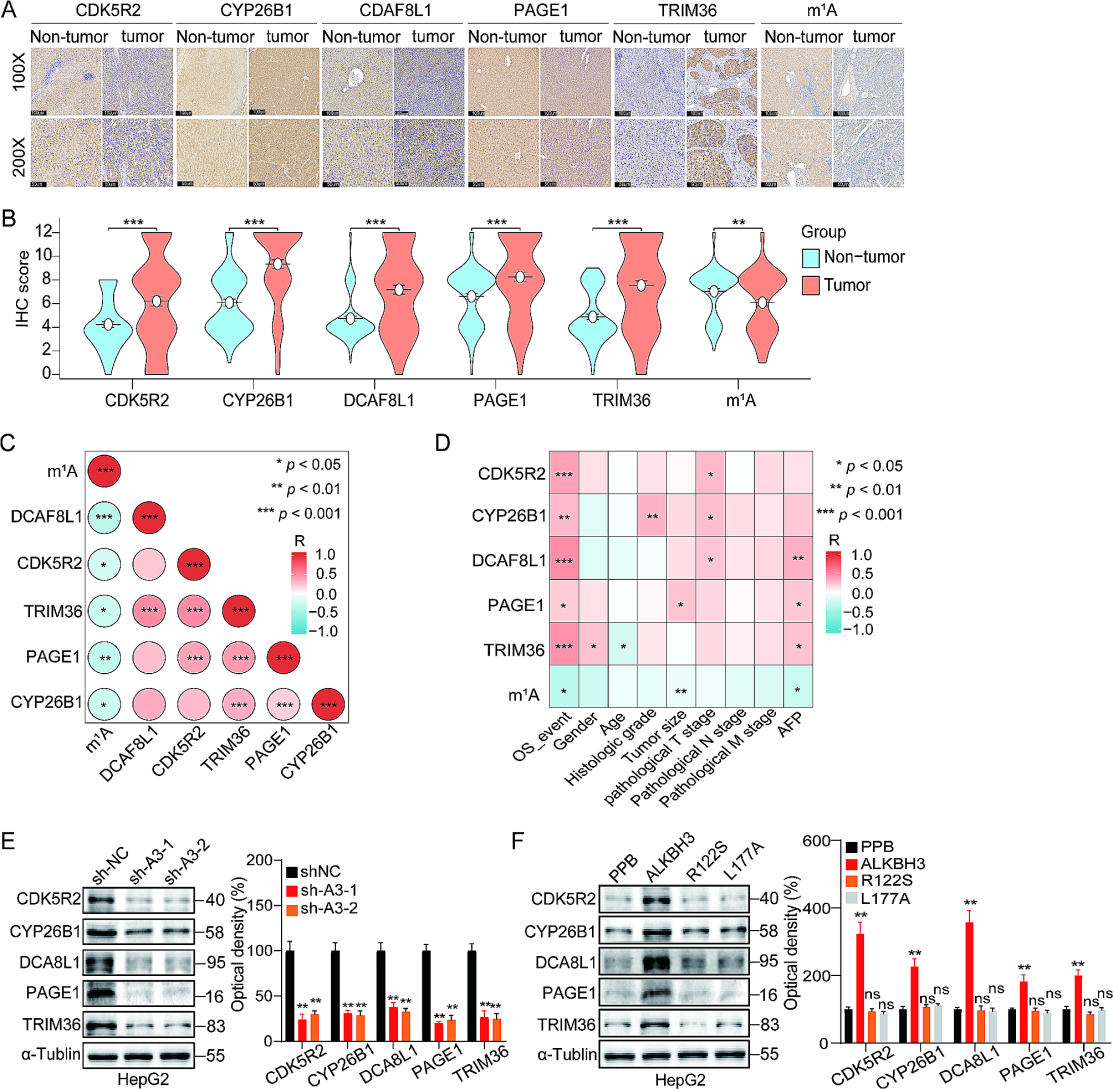
Analysis of the expression characteristics of the five differentially expressed genes (DEGs) in hepatocellular carcinoma (HCC) tissues. (**A** and **B**) Immunohistochemistry (IHC) analysis was performed to detect the expression of CYP26B1, CDK5R2, PAGE1, TRIM36, DCAF8L1, and m^1^A in HCC tissues (*n* = 90) and para-carcinoma tissues (*n* = 90; magnifications: 100× and 200×); (**C**) Heatmaps of correlations demonstrating the spectrum of relationships among targeting m^1^A, CYP26B1, CDK5R2, PAGE1, TRIM36, and DCAF8L1. The bar ranging from blue to red (− 1 to 1) represents negative to positive correlations, respectively; (**D**) Heatmaps of correlations demonstrating the spectrum of relationships among targeting CYP26B1, CDK5R2, PAGE1, TRIM36, DCAF8L1, m^1^A, and clinical features. The bar ranging from blue to red (− 1 to 1) represents negative to positive correlations, respectively; (**E**) Expression of TRIM36, CYP26B1, PAGE1, CDK5R2, and DCAF8L1 in sh-ALKBH3 and shNC HepG2 cells assessed through western blot (left) and quantitatively analyzed (right); (**F**) HepG2 cells were transfected with vector control, ALKBH3, ALKBH3-R122S, or ALKBH3-L177A constructs for 48 h, the expression of TRIM36, CYP26B1, PAGE1, CDK5R2, and DCAF8L1 were checked by western blot (left) and quantitatively analyzed (right); *p < 0.05, **p < 0.01, ***p < 0.001; ns, not significant

The results emphasized the impact of the five differentially expressed genes (DEGs) on the patient prognosis in cases of HCC. In order to further examine the effect in clinical application, the immunohistochemistry (IHC) technique was used to evaluate the expression levels of the five differentially expressed genes (TRIM36, CYP26B1, PAGE1, CDK5R2, and DCAF8L1) in 90 HCC tissues and 90 para-carcinoma tissues on a tissue microarray. Most HCC tissues exhibited elevated protein levels of CDK5R2, CYP26B1, DCAF8L1, PAGE1, and TRIM36 in comparison to para-carcinoma tissues, as indicated by the results. Conversely, the level of m^1^A modification decreased in most HCC tissues (Fig. 9A-B). Furthermore, to affirm the relationship between these five DEGs and m^1^A modification, an analysis of the expression correlation between CDK5R2, CYP26B1, DCAF8L1, PAGE1, TRIM36, and m^1^A modification in HCC tissues was conducted. As depicted in Fig. 9C, the m^1^A modification showed a significant negative correlation with these five DEGs (all *p* < 0.05).

Furthermore, to further identify the effects of the expression of the five genes on survival and prognosis, an integrative correlation analysis between the expression levels of the five DEGs and various clinical parameters such as OS rate, tumor grades, tumor size, tumor stages, distant metastasis status, and AFP level (the gold standard for HCC diagnosis) was conducted. This analysis identified TRIM36, CYP26B1, PAGE1, CDK5R2, and DCAF8L1 as a molecular signature associated with tumor aggressiveness (Fig. 9D). What’s more, knockdown of ALKBH3 significantly inhibited the expression of TRIM36, CYP26B1, PAGE1, CDK5R2, and DCAF8L1 in HepG2 cells (Fig. 9E). On the contrary, overexpression of ALKBH3 WT constructs, while not R122S or L177A mutant, up-regulated the expression of TRIM36, CYP26B1, PAGE1, CDK5R2, and DCAF8L1 in HepG2 cells (Fig. 9F). Collectively, these results highlight the potential of this five-DEG model, regulated by m^1^A modification and associated with autophagy, as a promising molecular signature for predicting HCC prognosis.

### Evaluating the diagnostic significance of the expression pattern of five differentially expressed genes (DEGs) in tissues from individuals diagnosed with hepatocellular carcinoma (HCC)

**Fig. 10:**
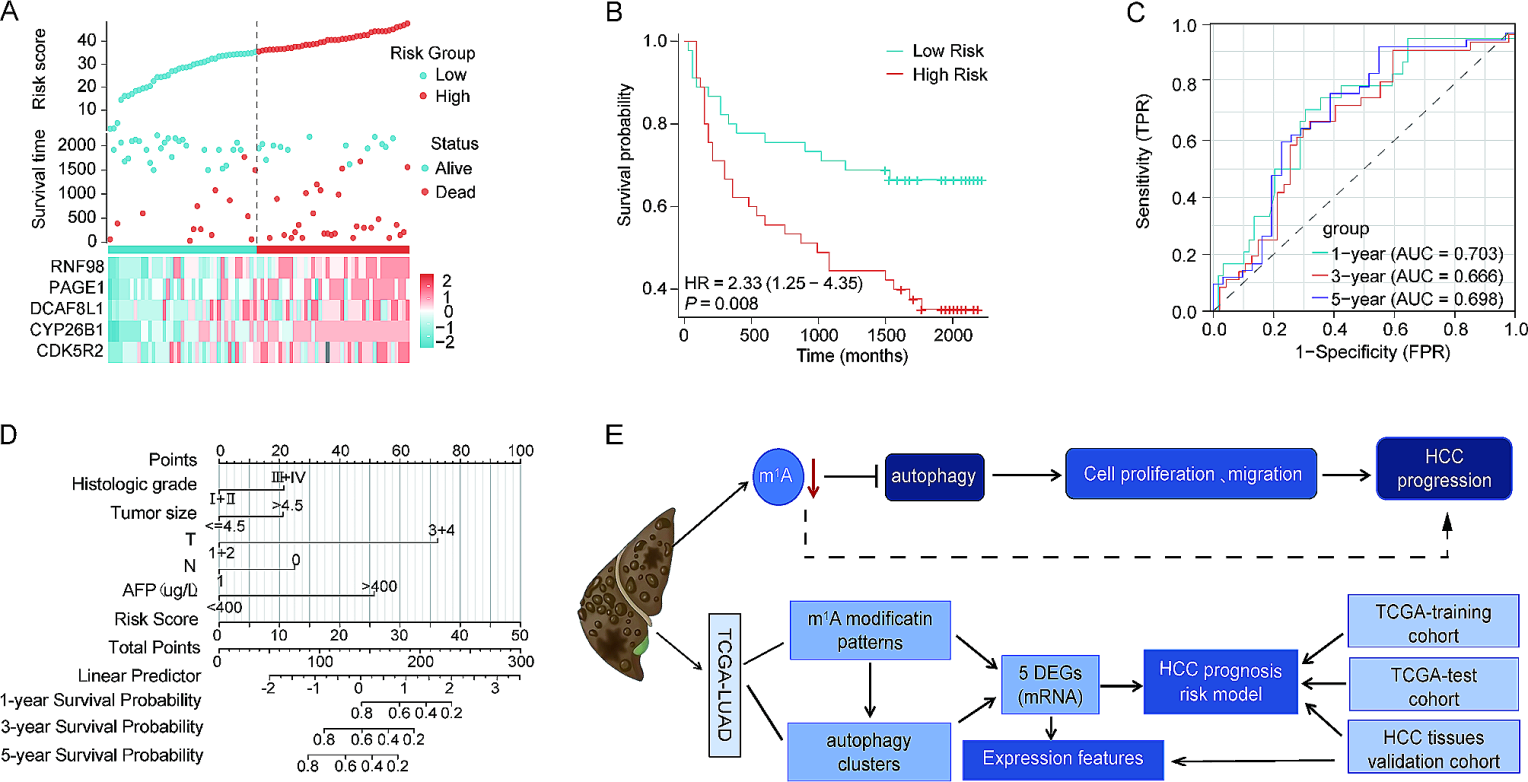
Analysis of the diagnostic value of the signature of the five differentially expressed genes (DEG) in tissues of hepatocellular carcinoma (HCC) patients. (**A**) Using tissue chips, HCC samples were divided into low-risk and high-risk categories. Survival time and survival status of each patient with HCC in tissue chips (top). Expression levels of TRIM36, CYP26B1, PAGE1, CDK5R2, and DCAF8L1 in HCC tissues from patients with high-risk and low-risk scores in tissue chips (bottom); (**B**) Analysis of Kaplan-Meier plots of overall survival rates for patients with HCC according to their risk scores; (**C**) The receiver operating characteristic curves show the diagnostic value of the risk model for patients with HCC at 1-, 3-, and 5-years after diagnosis; (**D**) Histologic grade, tumor size, T stage, N stage, alpha-fetoprotein (AFP), and risk score were used to construct the nomogram. (**E**) A flowchart to illustrate our research

Moreover, patients with HCC on tissue chips were also categorized into high-risk (*n* = 41) and low-risk (*n* = 41) score groups based on the risk score calculated by summing the risk coefficient from the five-gene model. Consistent with the results obtained for the training cohort and the test cohort, more deaths were reported among patients in the high-risk group, and the expression levels of CDK5R2, CYP26B1, DCAF8L1, PAGE1, and TRIM36 were notably higher in HCC tissues with high-risk scores (Fig. 10A). Kaplan–Meier curve analysis further validated the lower OS rate in the high-risk group (Fig. 10B). Additionally, ROC curve analysis revealed that the AUCs for predicting the 1-year, 3-year, and 5-year survival rates were 0.703, 0.666, and 0.698, respectively, in patients with HCC on tissue chips (Fig. 10C). Furthermore, a nomogram was constructed, and it displayed a high prognostic value for 1-year, 3-year, and 5-year survival rates (Fig. 10D). Altogether, this novel m^1^A-modification-related model associated with autophagy presents a promising prognostic signature for patients with HCC.

## Discussion

Epigenetic aberrations represent an emerging hallmark of tumorigenesis and progression [29]. The modification of RNA, specifically m^1^A RNA methylation, has received considerable focus in the field of epigenetics because of its numerous internal modifications and its connection to essential biological processes like RNA metabolism and protein translation [4, 30]. Accumulating evidence underscores the critical role of m^1^A modification in cancer development. Regulators associated with m^1^A have the potential to impact the advancement of cancer [10, 31–33]; additionally, the abundance of m^1^A and/or regulators connected to m^1^A could potentially be utilized as innovative biomarkers or targets for cancer treatment [27, 33–35]. Nevertheless, the extent and biological roles of m^1^A methylation in the advancement and prediction of HCC are still widely unexplored. Understanding the distinct roles of m^1^A modification patterns, the gene signatures associated with these patterns, and the biological events involved in their modulation, as well as changes in m^1^A modification levels in HCC, will enhance our understanding of m^1^A modification characteristics in HCC. This understanding will help guide more effective therapy strategies and prognostication.

The majority of patients with HCC often miss their ideal treatment opportunity due to delayed diagnosis [36, 37]. Recognizing the critical role of m^1^A in tumor progression and prognosis, a reliable prognostic signature based on five DEGs characterizing autophagy among the m^1^A modification patterns (CDK5R2, CYP26B1, DCAF8L1, PAGE1, and TRIM36) was initially established. The clinical usefulness of this study was validated in patients with HCC, indicating that this predictive pattern possesses significant diagnostic worth for 1-year, 3-year, and 5-year survival rates, the AUC values in the training cohort were 0.773, 0.699, 0.677. Models constructed based on m^6^A, m^7^G or m^5^C-related regulatory genes for the diagnosis in patients with HCC has been reported, the AUC values range from 0.61 to 0.74 [38–40]. However, so far, except for AFP, there is no single stand-alone biological indicator that has yet been identified as a biomarker for the diagnosis of HCC [41]. Despite AFP being considered the gold standard for HCC diagnosis and widely used in clinical practice, its limitations are evident, as many patients with advanced HCC are AFP-negative. The sensitivity and specificity of AFP diagnosis remain unsatisfactory [42]. This indicates that the prognostic signature holds high value in diagnosing HCC.

In this study, for the first time, we constructed a model for predicting the prognosis of HCC based on 5 DEGs (CDK5R2, CYP26B1, DCAF8L1, PAGE1, and TRIM36) of m^1^A modification correlated with autophagy. Studies have shown that CDK5R2 CYP26B1, DCAF8L1, PAGE1, and TRIM36 can be used to construct prognostic models or prognostic biomarker for HCC, rectal cancer, neuroblastoma, etc [43–47]. CDK5R2 is regulatory subunit 2 of Cyclin dependent kinase 5 (CDK5), it can interact with CDK5R1 and pericentrin (PCNT), plays a role in centriole engagement and microtubule nucleation. In HCC, vascular invasion may be related to the regulatory role of PART1 on CDK5R2 [48]. CYP26B1 is one of the main metabolic enzymes of tamoxifen, which can activate tamoxifen and make it effective. It is mainly expressed in liver and lowered CYP2D6 activity and altered protein expression in the tumor microenvironment reduce the risk of TT genotype-associated HCC [49]. DCAF8L1 is a member of the WD repeat protein family. Members of this family are involved in a variety of cellular processes, including cell cycle progression, signal transduction, apoptosis, and gene regulation. However, until now, its relationship with cancer progression was known limit. In the current study, we found that DCAF8L1 was significantly increased in HCC tissues campare to para-carcinoma tissues and positively associated with tumor aggressiveness, including OS rate, T stage and AFP level. PAGE1 belongs to a family of genes that are expressed in a variety of tumors but not in normal tissues, except for the testis. PAGE1 in serum may be an immunosuppressive biomarker for pancreatic ductal carcinoma [50]. TRIM36 is a member of the TRIM family, multiple alternatively spliced transcript variants that encode different protein isoforms have been described for this gene. TRIM36 can inhibit tumorigenesis through the Wnt/β-catenin pathway and promote caspase-dependent apoptosis in HCC [51]. Take together, these 5 DEGs play an important regulatory role in the malignant phenotype of tumors, cancer prognosis may benefit from a model based on this 5 DEGs.

Recently, the identification of changes in specific regulatory genes associated with m^1^A in HCC has established a definitive connection with clinicopathological characteristics and prognosis [52]. The essential role of m^1^A-related regulatory gene CNV in m^1^A regulation has been confirmed. To construct m^1^A modification patterns, genetic variation features of 10 m^1^A regulators in HCCs were identified using data from the TCGA database, including CNV alterations. YTHDF1, YTHDF3, and TRMT61B showed CNV alterations with higher amplification frequency, while TRMT61A, YTHDF1, YTHDF2, and YTHDC1 had a high deletion frequency. In 374 HCC samples, it was observed that the mRNA expression was elevated when there were higher copy numbers of m^1^A regulators, whereas deletions led to a decrease in mRNA expression (data not presented). However, compared with normal liver tissues, all 10 m^1^A regulators exhibited higher expression in HCC tissues, indicating that the expression alterations in m^1^A regulators, dynamically controlling m^1^A modification, may crucially regulate HCC progression.

By examining the expression of 10 m^1^A regulators, we were able to identify two separate clusters, namely cluster 1 and cluster 2, which displayed distinct patterns of m^1^A methylation modification. GSVA enrichment analysis unveiled significant differences in the expression of autophagy-related genes between cluster 1 and cluster 2. Accumulating evidence suggests that post-translational modifications (PTMs) and RNA methylation, such as the m^6^A-YTHDF3/FOXO3 mRNA axis [21], can influence autophagy induction in response to fasting or energy restriction [53]. In this context, a global increase in mRNA m^1^A methylation was observed, which was attributed to nutrient deficiency. The upregulated levels of m^1^A modification appear to activate autophagy, suggesting a potential regulatory role of m^1^A modification in autophagy processes. Moreover, the current study demonstrated that autophagy induced by mRNA m^1^A modification inhibits HCC cell proliferation. Hence, controlling m^1^A might represent a feasible strategy to regulate autophagic activity and effectively inhibit cell growth. This corroborates a double safeguard mechanism, as previously reported [27], involving the maintenance of ALKBH3 protein deletion and its dysregulated dynamic catalytic activity. This mechanism ensures elevated m^1^A levels, beneficial in impeding cancer cell proliferation and glycolysis. The regulation of autophagy in HCC cells through m^1^A warrants further exploration in subsequent studies, delving deeper into the underlying mechanisms.

In addition, the study also found that the m^1^A methylation level was lower in HCC tissues compared to paracancer tissues (Fig. 9B). The low level of m^1^A modification was positively associated with the malignancy of HCC, correlating positively with tumor size and OS (Fig. 9D). This indicates that a low level of m^1^A modification promotes HCC progression. Cancerous cells in solid tumors often encounter different internal and environmental disturbances that activate adaptive reactions, promoting the survival and advancement of cancer cells [54]. Data from our recent study have also shown that maintaining low levels of m^1^A may be a potential mechanism by which HCC adapts to survive under adverse conditions. This includes maintaining an appropriate level of autophagy required by cells. Any method that can increase the level of m^1^A methylation may prove an effective strategy for treating patients with HCC.

However, some limitations were also existed in our present study. The epigenetic regulatory relationship between m^1^A and autophagy was still known limited. Moreover, more experiments need to be performed to determine the regulatory mechanisms of 5 DEGs under autophagy.

## Conclusion

In summary (Fig. 10E), our work presents a novel m^1^A regulators model correlating with autophagy, predicting the prognosis of HCC. It demonstrates the regulatory mechanism between m^1^A mRNA modification and autophagy in this context. Notably, the variation in the reduced level of m^1^A modification in HCC should not be ignored, this could provide a potential target for anti-HCC treatment. Moreover, our findings will aid in further clarifying the molecular mechanism of mRNA m^1^A modification in HCC development.

### Electronic supplementary material

Below is the link to the electronic supplementary material.

Supplementary Material 1

Supplementary Material 2

Supplementary Material 3

## Data Availability

To acquire the experimental datasets utilized and analyzed in this study, please reach out to the corresponding authors and submit a reasonable request. The HCC training and test cohorts made use of RNA-seq data acquired from TCGA (https://portal.gdc.cancer.gov).

## Abbreviations

m^1^A: N1-methyladenosine
HCC: Hepatocellular carcinoma
TCGA-LIHC: The Cancer Genome Atlas-Liver Hepatocellular Carcinoma
DEGs: Differential expression genes
m^6^A: N6-methyladenosine
m^5^C: 5-methylcytosine
m^7^G: N7-methylguanosine
OS: Overall survival
CNV: Copy number variation
PCA: Principal component analysis
GSVA: Gene set variation analysis
COX: Cox proportional-hazards model
LASSO: Least absolute shrinkage and selection operator
ROC: Receiver operating characteristic curve
AUC: Area under curve
CDF: Cumulative distribution function
EBSS: Earle’s balanced salt solution
PTMs: Post-translational modifications
