# Supplementary material for: m1A regulator-mediated methylation modification patterns correlated with autophagy to predict the prognosis of hepatocellular carcinoma
Source: BMC Cancer. 2024 Apr 22;24:506. doi: 10.1186/s12885-024-12235-4 (PMC11034060; doi:10.1186/s12885-024-12235-4)
Supplement: Supplementary file 1 — Supplementary Material 1 [file 12885_2024_12235_MOESM1_ESM.docx]

Table S1 The autophagy related genes

| Autophagy related genes | | | | | | | | | | | | | | |
| --- | --- | --- | --- | --- | --- | --- | --- | --- | --- | --- | --- | --- | --- | --- |
| AMBRA1 | APOL1 | ARNT | ARSA | ARSB | ATF4 | ATF6 | ATG10 | ATG12 | ATG16L1 | ATG16L2 | ATG2A | ATG2B | ATG3 | ATG4A |
| ATG4B | ATG4C | ATG4D | ATG5 | ATG7 | ATG9A | ATG9B | ATIC | BAG1 | BAG3 | BAK1 | BAX | BCL2 | BCL2L1 | BECN1 |
| BID | BIRC5 | BIRC6 | BNIP1 | BNIP3 | BNIP3L | C12orf44 | C17orf88 | CALCOCO2 | CAMKK2 | CANX | CAPN1 | CAPN10 | CAPN2 | CAPNS1 |
| CASP1 | CASP3 | CASP4 | CASP8 | CCL2 | CCR2 | CD46 | CDKN1A | CDKN1B | CDKN2A | CFLAR | CHMP2B | CHMP4B | CLN3 | CTSB |
| CTSD | CTSL1 | CX3CL1 | CXCR4 | DAPK1 | DAPK2 | DDIT3 | DIRAS3 | DLC1 | DNAJB1 | DNAJB9 | DRAM1 | EDEM1 | EEF2 | EEF2K |
| EGFR | EIF2AK2 | EIF2AK3 | EIF2S1 | EIF4EBP1 | EIF4G1 | ERBB2 | ERN1 | ERO1L | FADD | FAM48A | FAS | FKBP1A | FKBP1B | FOS |
| FOXO1 | FOXO3 | GAA | GABARAP | GABARAPL | 1GABARAPL | 2GAPDH | GNAI3 | GNB2L1 | GOPC | GRID1 | GRID2 | HDAC1 | HDAC6 | HGS |
| HIF1A | HSP90AB1 | HSPA5 | HSPA8 | HSPB8 | IFNG | IKBKB | IKBKE | IL24 | IRGM | ITGA3 | ITGA6 | ITGB1 | ITGB4 | ITPR1 |
| GAA | GABARAP | GABARAPL | 1GABARAPL | 2GAPDH | GNAI3 | GNB2L1 | GOPC | GRID1 | GRID2 | KIAA0226 | KIAA0652 | KIAA0831 | KIF5B | KLHL24 |
| LAMP1 | LAMP2 | MAP1LC3A | MAP1LC3B | MAP1LC3C | MAP2K7 | MAPK1 | MAPK3 | MAPK8 | MAPK8IP1 | MAPK9 | MBTPS2 | MLST8 | MTMR14 | MTOR |
| MYC | NAF1 | NAMPT | NBR1 | NCKAP1 | NFE2L2 | NFKB1 | NKX2-3 | NLRC4 | NPC1 | NRG1 | NRG2 | NRG3 | P4HB | PARK2 |
| PARP1 | PEA15 | PELP1 | PEX14 | PEX3 | PIK3C3 | PIK3R4 | PINK1 | PPP1R15A | PRKAB1 | PRKAR1A | PRKCD | PRKCQ | PTEN | PTK6 |
| RAB11A | RAB1A | RAB24 | RAB33B | RAB5A | RAB7A | RAC1 | RAF1 | RB1 | RB1CC1 | RELA | RGS19 | RHEB | RPS6KB1 | RPTOR |
| SAR1A | SERPINA1 | SESN2 | SH3GLB1 | SIRT1 | SIRT2 | SPHK1 | SPNS1 | SQSTM1 | ST13 | STK11 | TBK1 | TM9SF1 | TMEM49 | TMEM74 |
| TNFSF10 | TP53 | TP53INP2 | TP63 | TP73 | TSC1 | TSC2 | TUSC1 | ULK1 | ULK2 | ULK3 | USP10 | UVRAG | VAMP3 | VAMP7 |
| VEGFA | WDFY3 | WDR45 | WDR45L | WIPI1 | WIPI2 | ZFYVE1 |  |  |  |  |  |  |  |  |
