# Supplementary material for: m1A regulator-mediated methylation modification patterns correlated with autophagy to predict the prognosis of hepatocellular carcinoma
Source: BMC Cancer. 2024 Apr 22;24:506. doi: 10.1186/s12885-024-12235-4 (PMC11034060; doi:10.1186/s12885-024-12235-4)
Supplement: Supplementary file 2 — Supplementary Material 2 [file 12885_2024_12235_MOESM2_ESM.doc]

Table S2

Patients’ ID of training set (n=186) and validation set (n=188) in TCGA-LIHC dataset

|  | ID |
| --- | --- |
| TCGA-train | TCGA-2Y-A9GU-01A TCGA-2Y-A9GV-01A TCGA-2Y-A9GW-01A TCGA-2Y-A9GY-01A TCGA-2Y-A9H0-01A TCGA-2Y-A9H4-01A TCGA-2Y-A9H7-01A TCGA-2Y-A9HA-01A  TCGA-2Y-A9HB-01A TCGA-4R-AA8I-01A TCGA-5C-A9VG-01A TCGA-BC-A10Q-01A TCGA-BC-A10U-01A TCGA-BC-A10W-01A TCGA-BC-A10Z-01A TCGA-BC-A110-01A TCGA-BC-A112-01A TCGA-BC-A217-01A TCGA-BC-A3KF-01A TCGA-BC-A3KG-01A TCGA-BC-A5W4-01A TCGA-BC-A69I-01A TCGA-BC-A8YO-01A TCGA-BD-A2L6-01A TCGA-BD-A3EP-01A TCGA-BW-A5NO-01A TCGA-CC-5259-01A TCGA-CC-5261-01A TCGA-CC-5262-01A TCGA-CC-5263-01A TCGA-CC-5264-01A TCGA-CC-A1HT-01A TCGA-CC-A3M9-01A TCGA-CC-A3MA-01A TCGA-CC-A3MC-01A TCGA-CC-A5UD-01A TCGA-CC-A7IF-01A TCGA-CC-A7IG-01A TCGA-CC-A7IH-01A TCGA-CC-A7II-01A TCGA-CC-A7IJ-01A TCGA-CC-A7IK-01A TCGA-CC-A8HS-01A TCGA-CC-A8HT-01A TCGA-CC-A9FS-01A TCGA-CC-A9FU-01A TCGA-CC-A9FV-01A TCGA-CC-A9FW-01A  TCGA-DD-A114-01A TCGA-DD-A118-01A TCGA-DD-A119-01A TCGA-DD-A11A-01A TCGA-DD-A11B-01A TCGA-DD-A11C-01A TCGA-DD-A11D-01A TCGA-DD-A1EE-01A TCGA-DD-A1EF-01A TCGA-DD-A1EI-01A TCGA-DD-A1EK-01A TCGA-DD-A1EL-01A TCGA-DD-A39Y-01A TCGA-DD-A39Z-01A TCGA-DD-A3A2-01A TCGA-DD-A3A8-01A TCGA-DD-A4NA-01A TCGA-DD-A4ND-01A TCGA-DD-A4NF-01A TCGA-DD-A4NH-01A TCGA-DD-A4NJ-01A TCGA-DD-A4NN-01A TCGA-DD-A4NQ-01A TCGA-DD-A73A-01A TCGA-DD-A73B-01A TCGA-DD-A73E-01A TCGA-DD-A73G-01A TCGA-DD-AAC8-01A TCGA-DD-AAC9-01A TCGA-DD-AACE-01A TCGA-DD-AACF-01A TCGA-DD-AACG-01A TCGA-DD-AACH-01A TCGA-DD-AACK-01A TCGA-DD-AACL-01A TCGA-DD-AACM-01A TCGA-DD-AACN-01A TCGA-DD-AACP-01A TCGA-DD-AACS-01A TCGA-DD-AACV-01A TCGA-DD-AACW-01A TCGA-DD-AACZ-01A TCGA-DD-AAD0-01A TCGA-DD-AADC-01A TCGA-DD-AADD-01A TCGA-DD-AADE-01A TCGA-DD-AADJ-01A TCGA-DD-AADL-01A TCGA-DD-AADM-01A TCGA-DD-AADP-01A TCGA-DD-AADQ-01A TCGA-DD-AADW-01A TCGA-DD-AADY-01A TCGA-DD-AAE1-01A TCGA-DD-AAE4-01A TCGA-DD-AAE6-01A TCGA-DD-AAE9-01A TCGA-DD-AAEA-01A TCGA-DD-AAEB-01A TCGA-DD-AAEE-01A TCGA-DD-AAVS-01A TCGA-DD-AAVU-01A TCGA-DD-AAW3-01A TCGA-ED-A4XI-01A TCGA-ED-A66X-01A TCGA-ED-A66Y-01A TCGA-ED-A7PX-01A TCGA-ED-A7PZ-01A TCGA-ED-A7XO-01A TCGA-ED-A8O5-01A TCGA-ED-A97K-01A TCGA-EP-A12J-01A TCGA-EP-A2KA-01A TCGA-EP-A2KB-01A TCGA-EP-A2KC-01A TCGA-EP-A3JL-01A TCGA-EP-A3RK-01A TCGA-FV-A23B-01A TCGA-FV-A3I0-01A TCGA-FV-A3I1-01A TCGA-FV-A3R2-01A TCGA-FV-A3R3-01A TCGA-FV-A4ZQ-01A TCGA-G3-A25S-01A TCGA-G3-A25T-01A TCGA-G3-A25V-01A TCGA-G3-A25Y-01A TCGA-G3-A3CG-01A TCGA-G3-A3CH-01A TCGA-G3-A3CI-01A TCGA-G3-A5SJ-01A TCGA-G3-A7M5-01A TCGA-G3-A7M6-01A TCGA-G3-A7M7-01A TCGA-G3-A7M8-01A TCGA-G3-A7M9-01A TCGA-G3-AAV2-01A TCGA-G3-AAV3-01A TCGA-G3-AAV5-01A TCGA-G3-AAV6-01A TCGA-G3-AAV7-01A TCGA-GJ-A6C0-01A TCGA-GJ-A9DB-01A TCGA-HP-A5MZ-01A TCGA-K7-A5RF-01A TCGA-K7-A5RG-01A TCGA-K7-AAU7-01A TCGA-KR-A7K0-01A TCGA-LG-A6GG-01A TCGA-LG-A9QD-01A TCGA-MI-A75C-01A TCGA-MI-A75E-01A TCGA-MI-A75H-01A TCGA-MR-A520-01A TCGA-MR-A8JO-01A TCGA-QA-A7B7-01A TCGA-RC-A6M3-01A TCGA-RC-A6M5-01A TCGA-RC-A6M6-01A TCGA-RC-A7SB-01A TCGA-RC-A7SH-01A TCGA-RC-A7SK-01A TCGA-RG-A7D4-01A TCGA-T1-A6J8-01A TCGA-UB-A7MD-01A TCGA-UB-A7ME-01A TCGA-UB-A7MF-01A TCGA-UB-AA0U-01A TCGA-UB-AA0V-01A TCGA-WJ-A86L-01A TCGA-WQ-A9G7-01A TCGA-WX-AA44-01A TCGA-XR-A8TF-01A TCGA-YA-A8S7-01A TCGA-ZP-A9CY-01A TCGA-ZP-A9D0-01A TCGA-ZP-A9D2-01A TCGA-ZP-A9D4-01A |
| TCGA-test | TCGA-BC-4072-01B TCGA-BC-4073-01B TCGA-2Y-A9GS-01A TCGA-2Y-A9GT-01A TCGA-2Y-A9GX-01A TCGA-2Y-A9GZ-01A TCGA-2Y-A9H1-01A TCGA-2Y-A9H2-01A TCGA-2Y-A9H3-01A TCGA-2Y-A9H5-01A TCGA-2Y-A9H6-01A TCGA-2Y-A9H8-01A TCGA-2Y-A9H9-01A TCGA-3K-AAZ8-01A TCGA-5C-A9VH-01A TCGA-5C-AAPD-01A TCGA-5R-AA1C-01A TCGA-5R-AA1D-01A TCGA-5R-AAAM-01A TCGA-BC-A10R-01A TCGA-BC-A10S-01A TCGA-BC-A10T-01A TCGA-BC-A10X-01A TCGA-BC-A10Y-01A TCGA-BC-A216-01A TCGA-BC-A69H-01A TCGA-BD-A3ER-01A TCGA-CC-5258-01A TCGA-CC-5260-01A TCGA-CC-A123-01A TCGA-CC-A3MB-01A TCGA-CC-A5UC-01A TCGA-CC-A5UE-01A TCGA-CC-A7IE-01A TCGA-CC-A7IL-01A TCGA-CC-A8HU-01A TCGA-CC-A8HV-01A TCGA-DD-A113-01A TCGA-DD-A115-01A TCGA-DD-A116-01A TCGA-DD-A1E9-01A TCGA-DD-A1EA-01A TCGA-DD-A1EB-01A TCGA-DD-A1EC-01A TCGA-DD-A1ED-01A TCGA-DD-A1EG-01A TCGA-DD-A1EH-01A TCGA-DD-A1EJ-01A TCGA-DD-A39V-01A TCGA-DD-A39W-01A TCGA-DD-A39X-01A TCGA-DD-A3A0-01A TCGA-DD-A3A1-01A TCGA-DD-A3A3-01A TCGA-DD-A3A4-01A TCGA-DD-A3A5-01A TCGA-DD-A3A6-01A TCGA-DD-A3A7-01A  TCGA-DD-A3A9-01A TCGA-DD-A4NB-01A TCGA-DD-A4NE-01A TCGA-DD-A4NG-01A TCGA-DD-A4NI-01A TCGA-DD-A4NK-01A TCGA-DD-A4NL-01A TCGA-DD-A4NO-01A TCGA-DD-A4NP-01A TCGA-DD-A4NR-01A TCGA-DD-A4NS-01A TCGA-DD-A4NV-01A TCGA-DD-A73C-01A TCGA-DD-A73D-01A TCGA-DD-A73F-01A TCGA-DD-AA3A-01A TCGA-DD-AACA-01A TCGA-DD-AACB-01A TCGA-DD-AACC-01A TCGA-DD-AACD-01A TCGA-DD-AACI-01A TCGA-DD-AACJ-01A TCGA-DD-AACO-01A TCGA-DD-AACQ-01A TCGA-DD-AACT-01A TCGA-DD-AACU-01A TCGA-DD-AACX-01A TCGA-DD-AACY-01A TCGA-DD-AAD1-01A TCGA-DD-AAD2-01A TCGA-DD-AAD3-01A TCGA-DD-AAD5-01A TCGA-DD-AAD6-01A TCGA-DD-AAD8-01A TCGA-DD-AADA-01A TCGA-DD-AADB-01A TCGA-DD-AADF-01A TCGA-DD-AADG-01A TCGA-DD-AADI-01A TCGA-DD-AADK-01A TCGA-DD-AADN-01A TCGA-DD-AADO-01A TCGA-DD-AADR-01A TCGA-DD-AADS-01A TCGA-DD-AADU-01A TCGA-DD-AADV-01A TCGA-DD-AAE0-01A TCGA-DD-AAE2-01A TCGA-DD-AAE3-01A TCGA-DD-AAE7-01A TCGA-DD-AAE8-01A TCGA-DD-AAED-01A TCGA-DD-AAEG-01A TCGA-DD-AAEH-01A TCGA-DD-AAEI-01A TCGA-DD-AAEK-01A TCGA-DD-AAVP-01A TCGA-DD-AAVQ-01A TCGA-DD-AAVR-01A TCGA-DD-AAVV-01A TCGA-DD-AAVW-01A TCGA-DD-AAVX-01A TCGA-DD-AAVY-01A TCGA-DD-AAVZ-01A TCGA-DD-AAW0-01A TCGA-DD-AAW1-01A TCGA-DD-AAW2-01A TCGA-ED-A459-01A TCGA-ED-A5KG-01A TCGA-ED-A627-01A TCGA-ED-A7PY-01A TCGA-ED-A7XP-01A TCGA-ED-A82E-01A TCGA-ED-A8O6-01A TCGA-EP-A26S-01A TCGA-ES-A2HS-01A TCGA-ES-A2HT-01A TCGA-FV-A2QQ-01A TCGA-FV-A2QR-01A TCGA-FV-A495-01A TCGA-FV-A496-01A TCGA-FV-A4ZP-01A TCGA-G3-A25U-01A TCGA-G3-A25W-01A TCGA-G3-A25X-01A TCGA-G3-A25Z-01A TCGA-G3-A3CJ-01A TCGA-G3-A3CK-01A TCGA-G3-A5SI-01A TCGA-G3-A5SK-01A TCGA-G3-A5SL-01A TCGA-G3-A5SM-01A TCGA-G3-A6UC-01A TCGA-G3-AAUZ-01A TCGA-G3-AAV0-01A TCGA-G3-AAV1-01A TCGA-G3-AAV4-01A TCGA-GJ-A3OU-01A TCGA-HP-A5N0-01A TCGA-K7-A6G5-01A TCGA-KR-A7K2-01A TCGA-KR-A7K7-01A TCGA-KR-A7K8-01A TCGA-LG-A9QC-01A TCGA-MI-A75G-01A TCGA-MI-A75I-01A TCGA-NI-A4U2-01A TCGA-NI-A8LF-01A TCGA-O8-A75V-01A TCGA-PD-A5DF-01A TCGA-RC-A6M4-01A TCGA-RC-A7S9-01A TCGA-RC-A7SF-01A TCGA-UB-A7MA-01A TCGA-UB-A7MB-01A TCGA-UB-A7MC-01A TCGA-WQ-AB4B-01A TCGA-WX-AA46-01A TCGA-WX-AA47-01A TCGA-XR-A8TC-01A TCGA-XR-A8TD-01A TCGA-XR-A8TE-01A TCGA-XR-A8TG-01A TCGA-ZP-A9CV-01A TCGA-ZP-A9CZ-01A TCGA-ZP-A9D1-01A TCGA-ZS-A9CD-01A TCGA-ZS-A9CE-01A TCGA-ZS-A9CF-01A TCGA-ZS-A9CG-01A |
|  |  |
