# Supplementary material for: m1A regulator-mediated methylation modification patterns correlated with autophagy to predict the prognosis of hepatocellular carcinoma
Source: BMC Cancer. 2024 Apr 22;24:506. doi: 10.1186/s12885-024-12235-4 (PMC11034060; doi:10.1186/s12885-024-12235-4)
Supplement: Supplementary file 3 — Supplementary Material 3 [file 12885_2024_12235_MOESM3_ESM.docx]

Figure 3A.


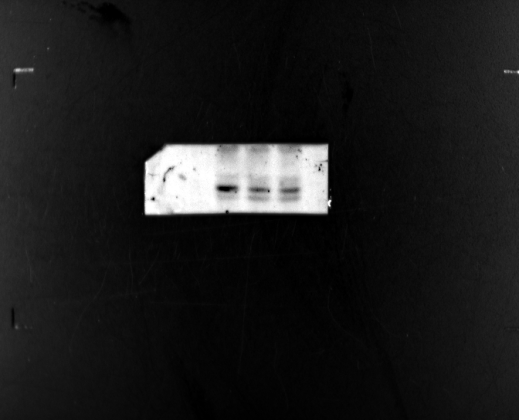

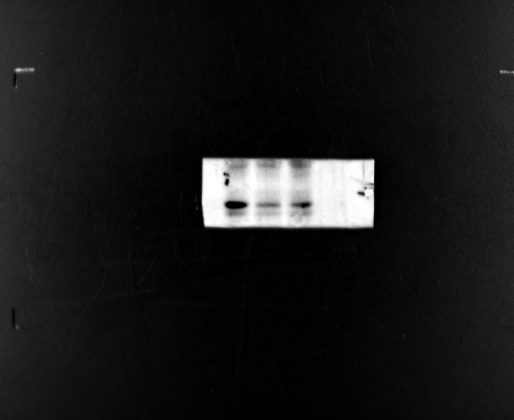

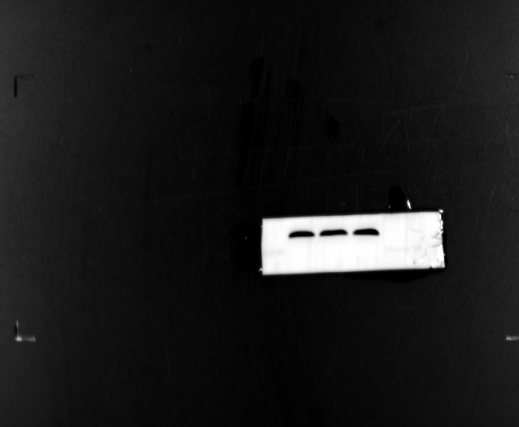

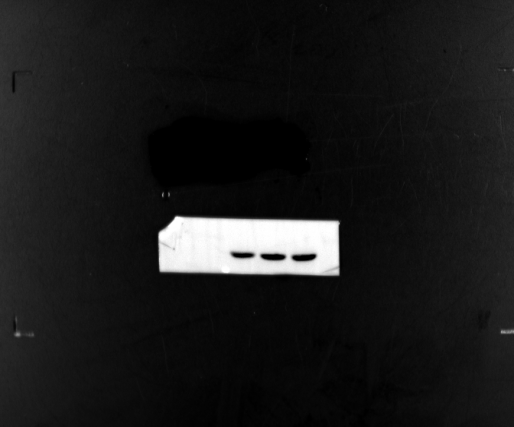


a-Tublin

ALKBH3

ALKBH3

HepG2

QGY

HepG2

QGY

a-Tublin

Figure 3B.


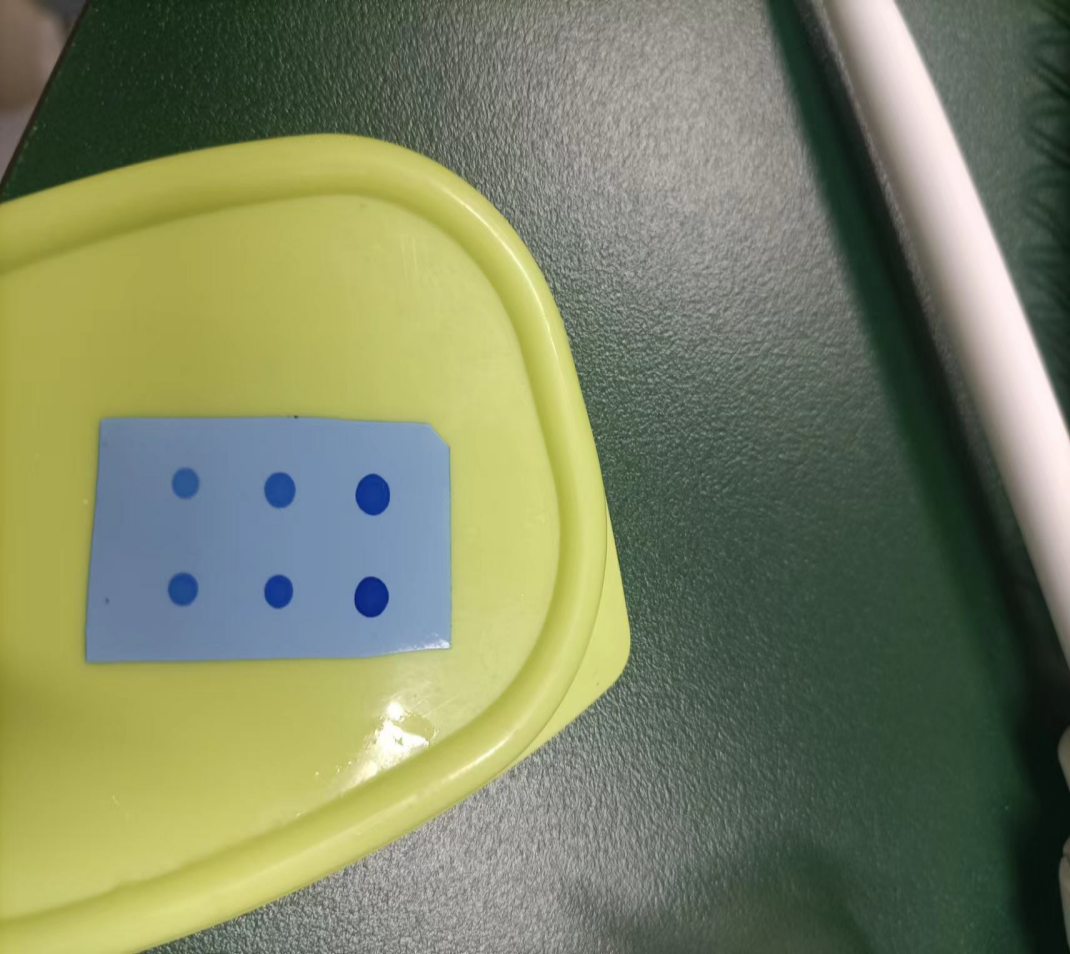

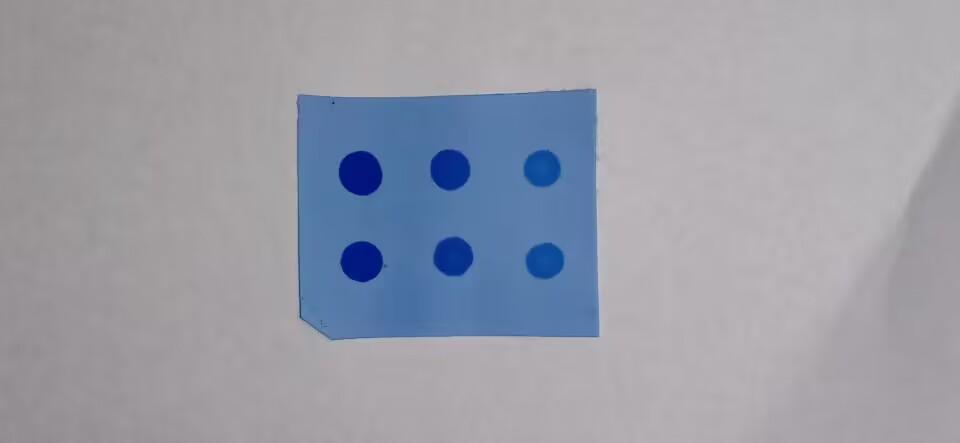


anti-m^1^A

input

QGY

HepG2


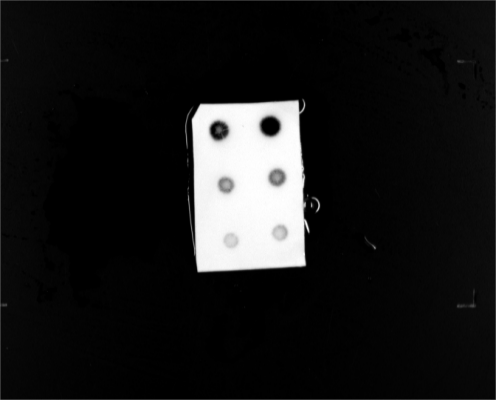

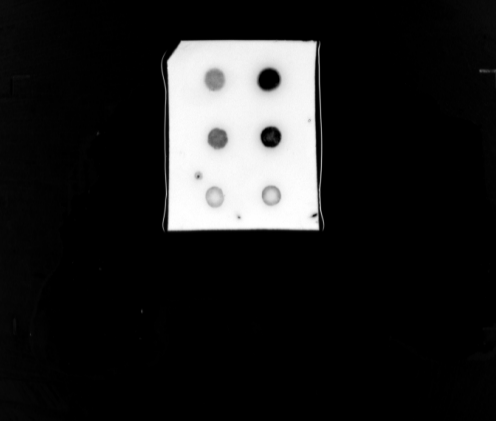


Figure 3D.

sh-NC

sh-A3

HepG2

QGY


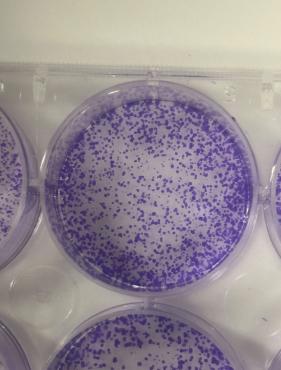

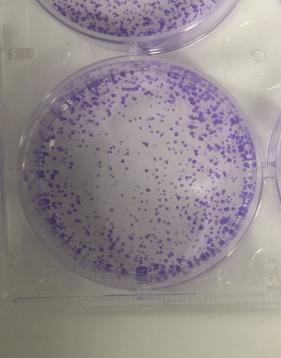

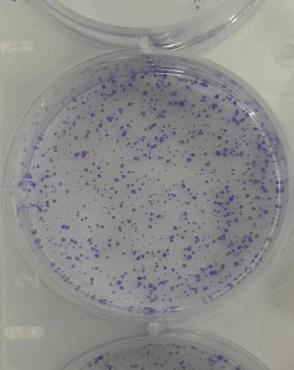

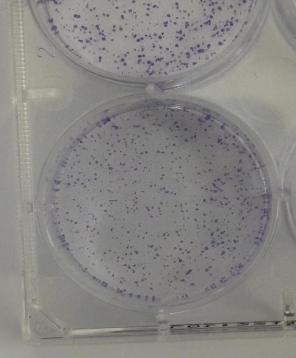

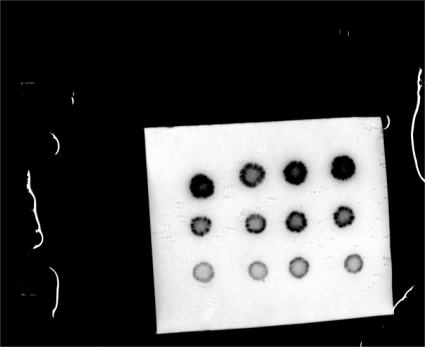

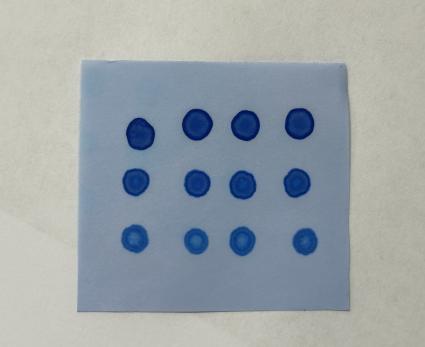


anti-m^1^A

input

Figure 3F.


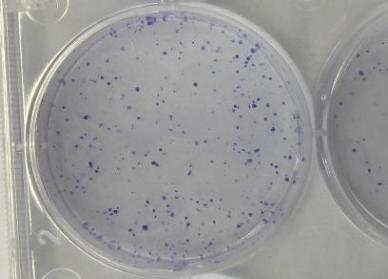

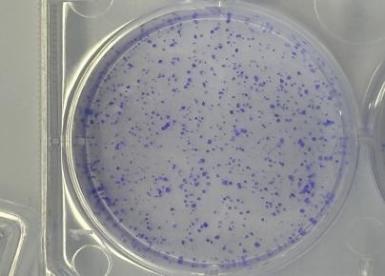

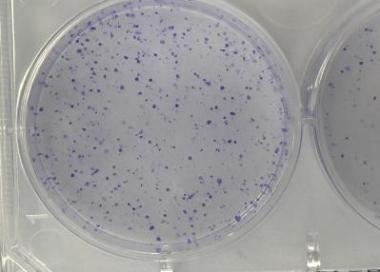


PPB

ALKBH3


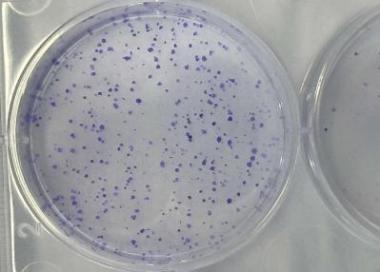


R122S

L177A

Figure 3H.

Figure 5A.

a-tublin


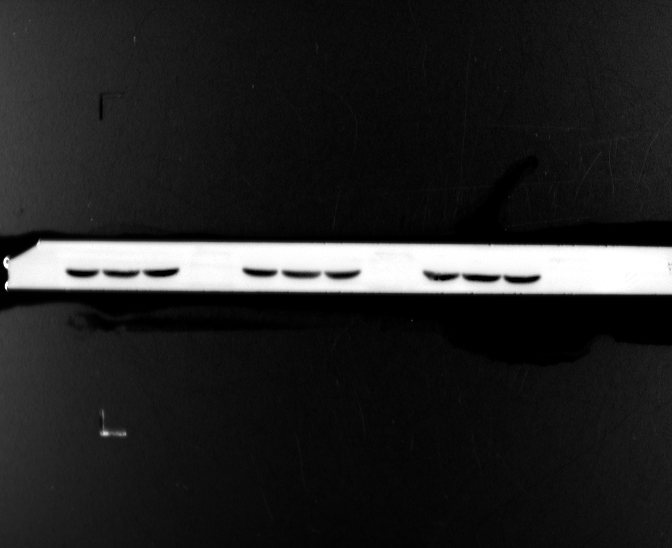


a-tublin


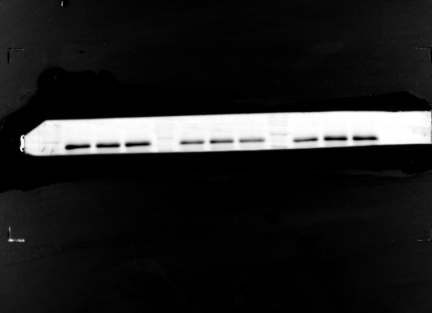

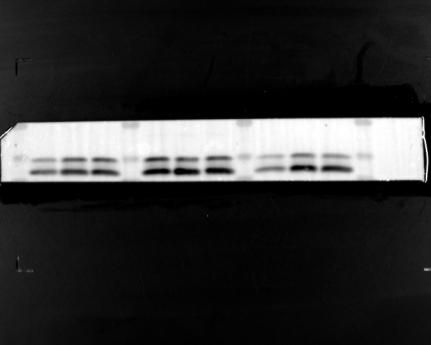


Beclin1

QGY

QGY

LC3

HepG2

a-Tublin

QGY

HepG2


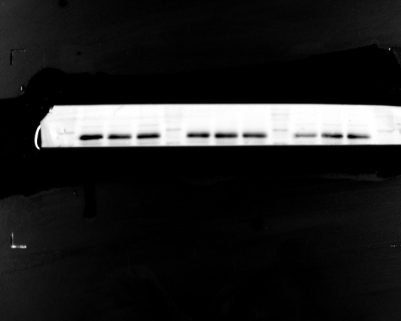


Beclin1

HepG2


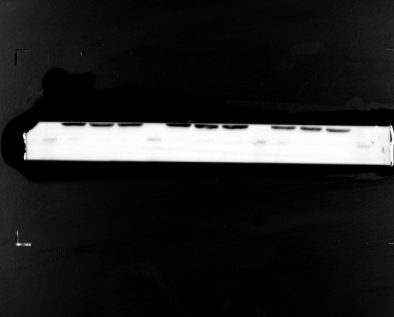


a-Tublin

HepG2


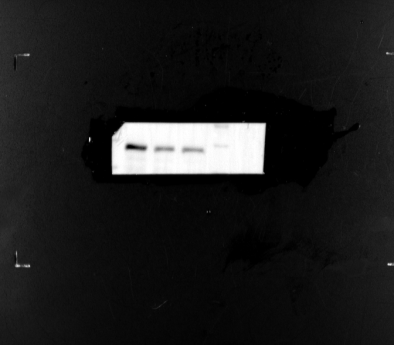


Beclin1

p62


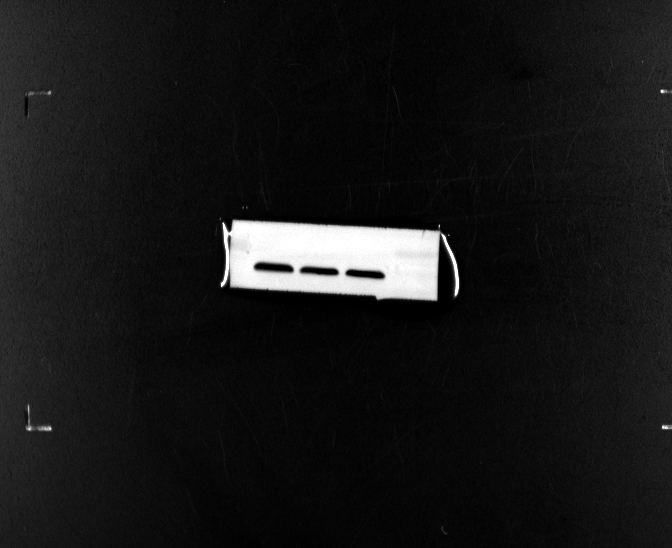

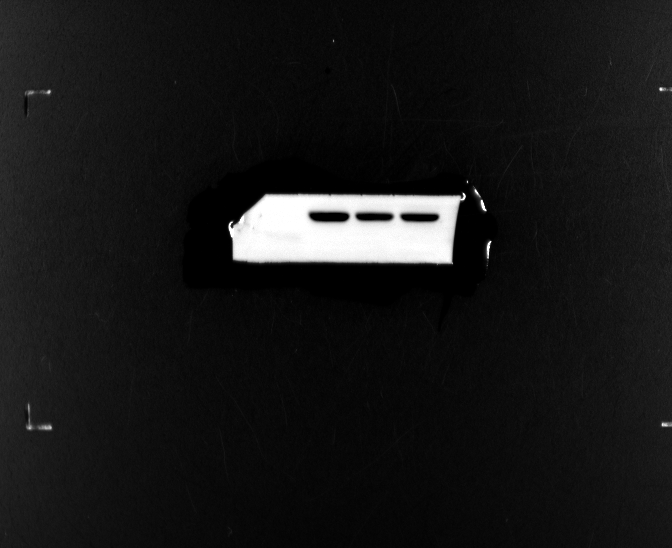


a-Tublin


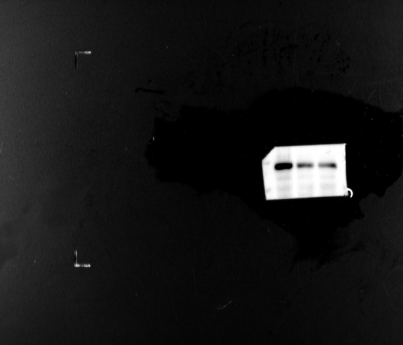


p62

a-Tublin

HepG2

HepG2

QGY

QGY

Figure 5C.


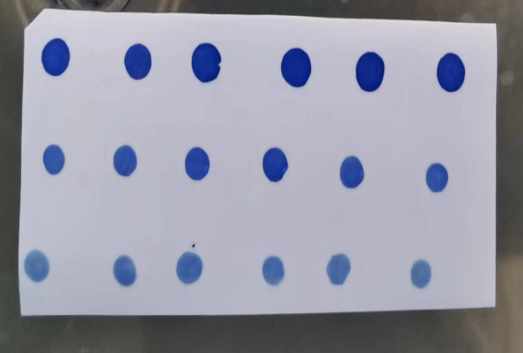


anti-m^1^A

input


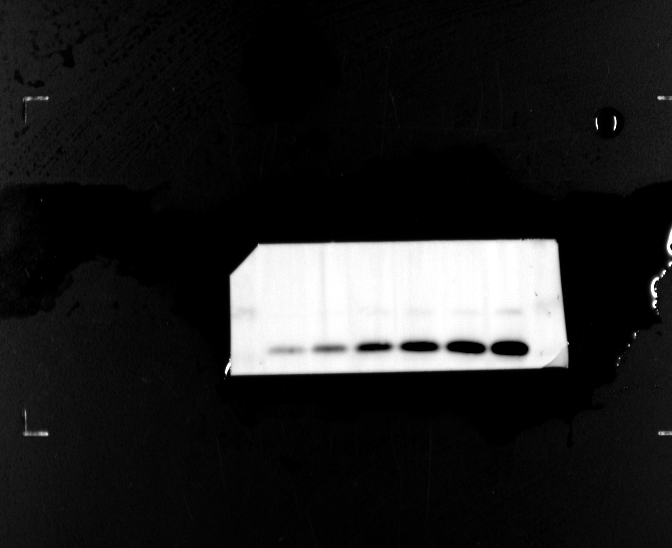

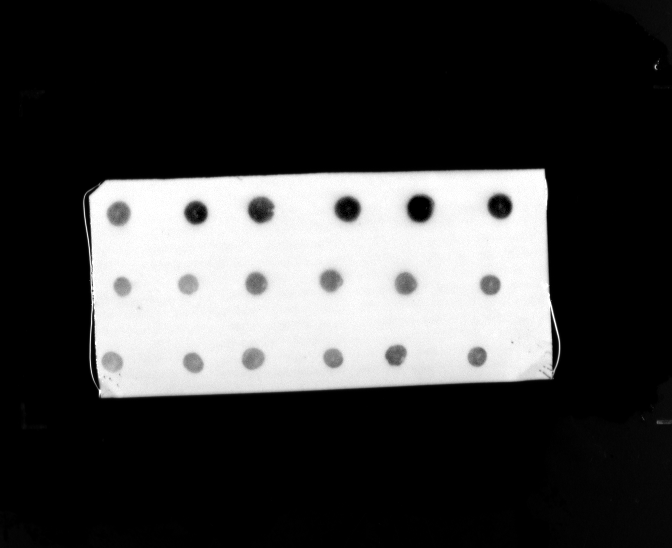


LC3


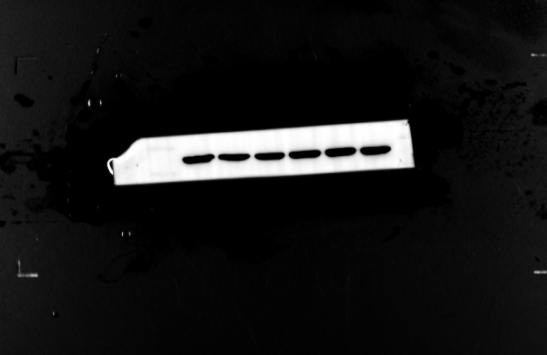


a-Tublin

Figure 5D.


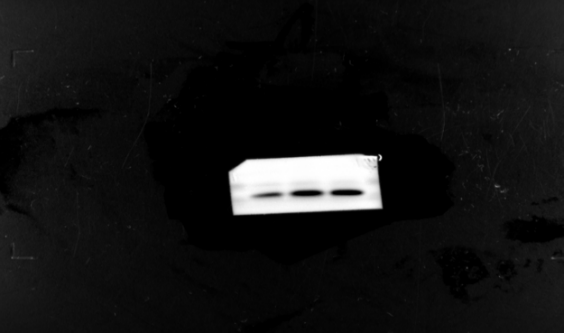


LC3


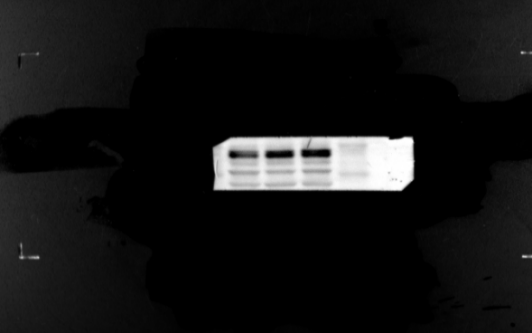


Beclin1


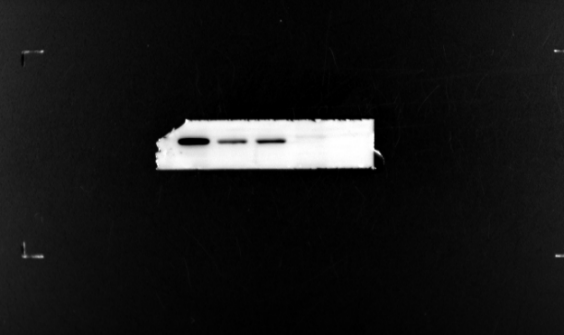


p62


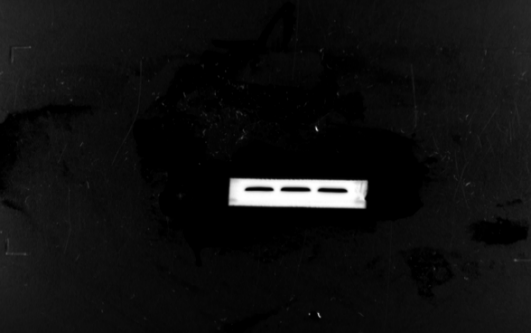


a-Tublin

Figure 5E.


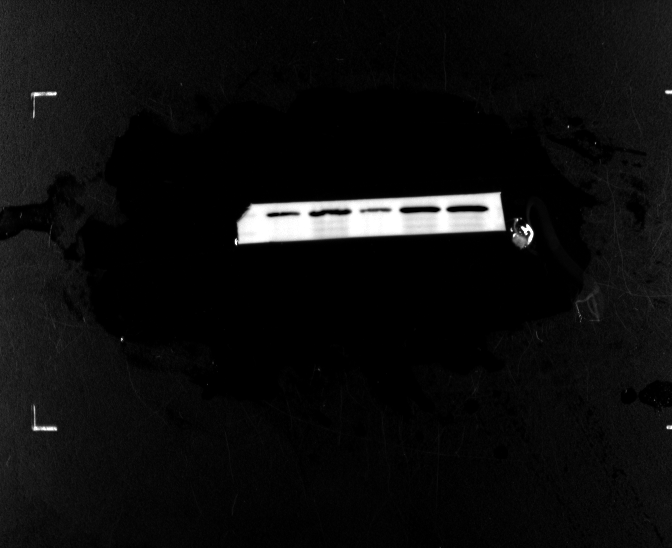

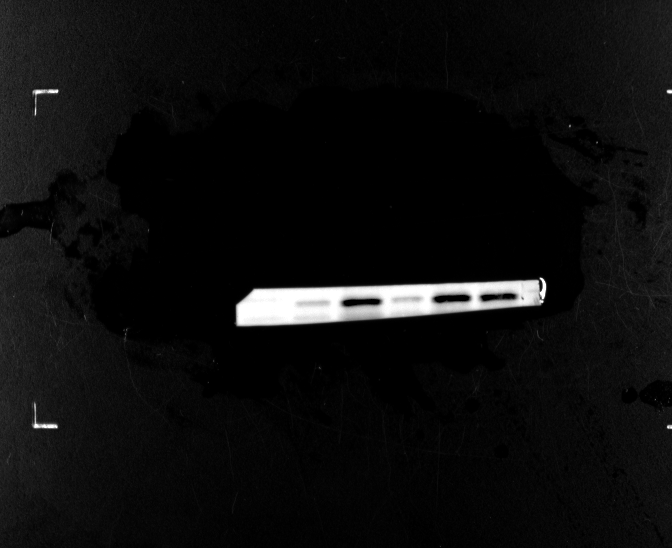

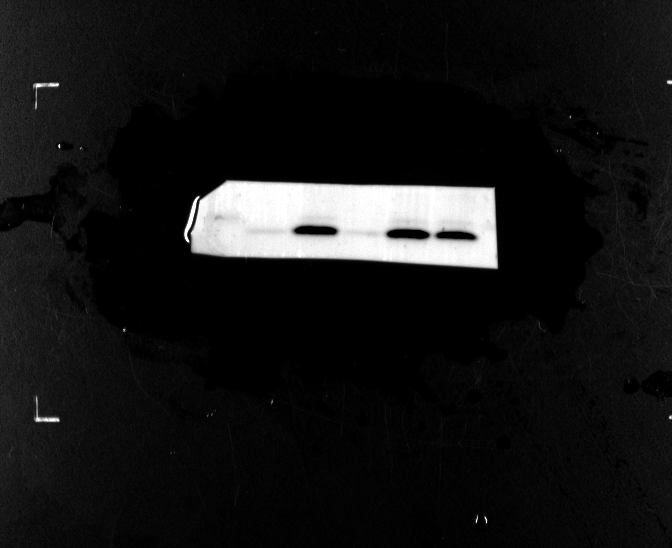

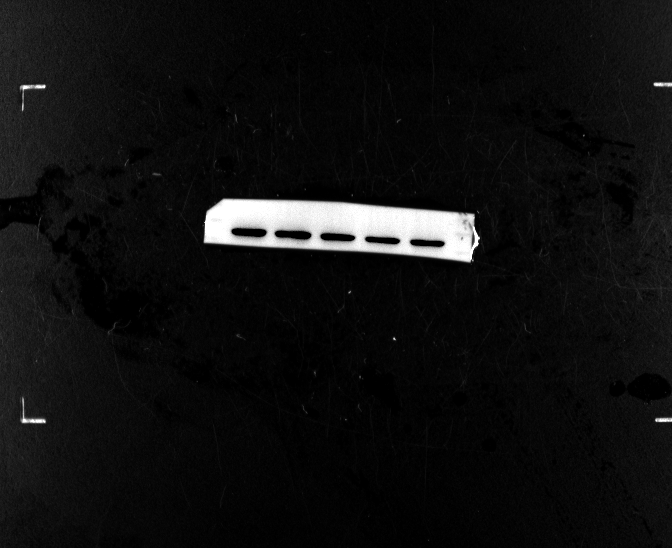


p62

Beclin1

LC3

a-Tublin

LC3

Beclin1

P62

a-Tublin

Figure 5G.


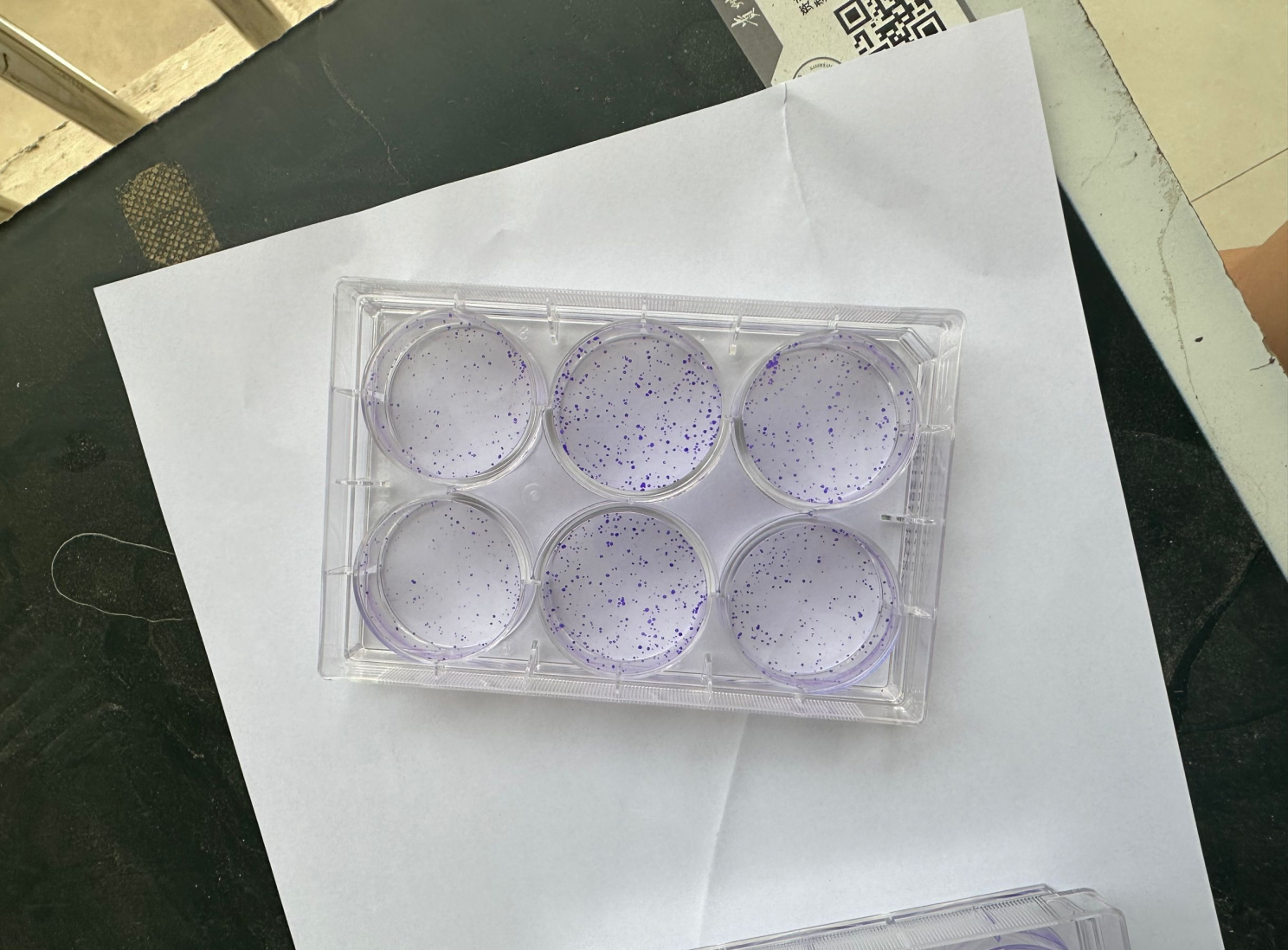


Con

EBSS

EBSS+A3

Figure 9E.


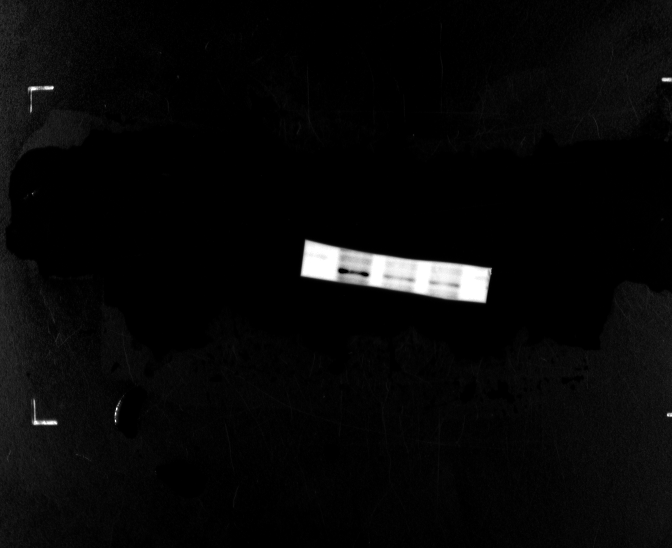


CDK5R2


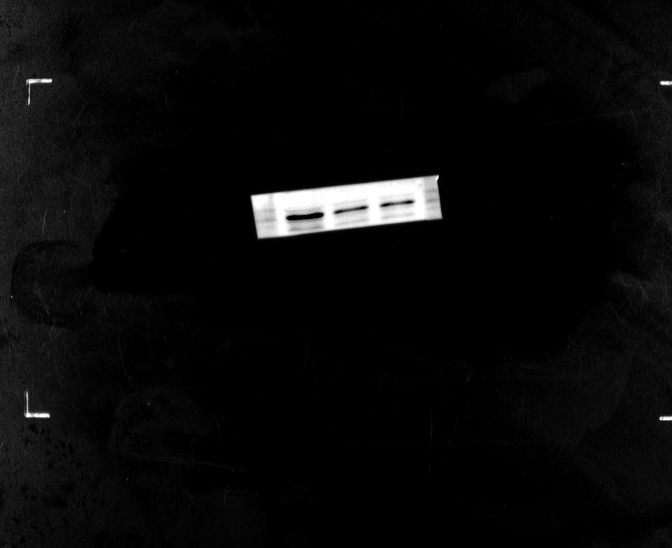


CYP26B1


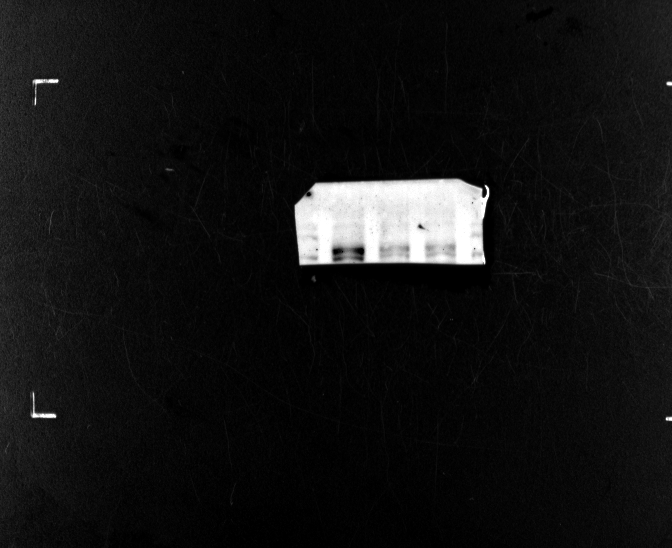


DCAF8L1


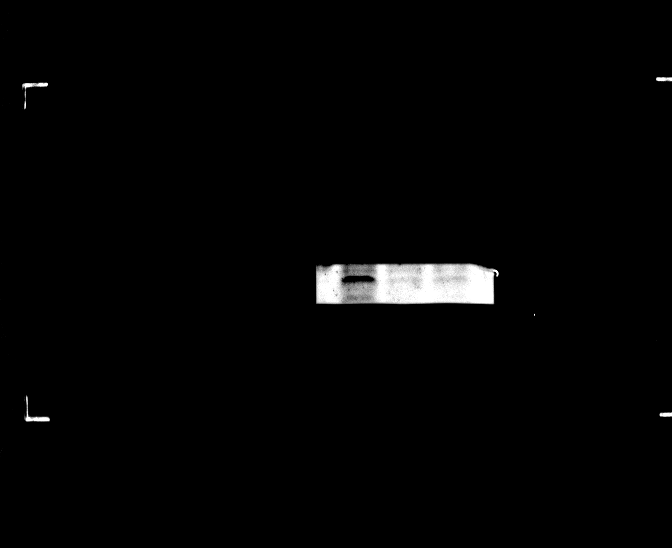


PAGE1


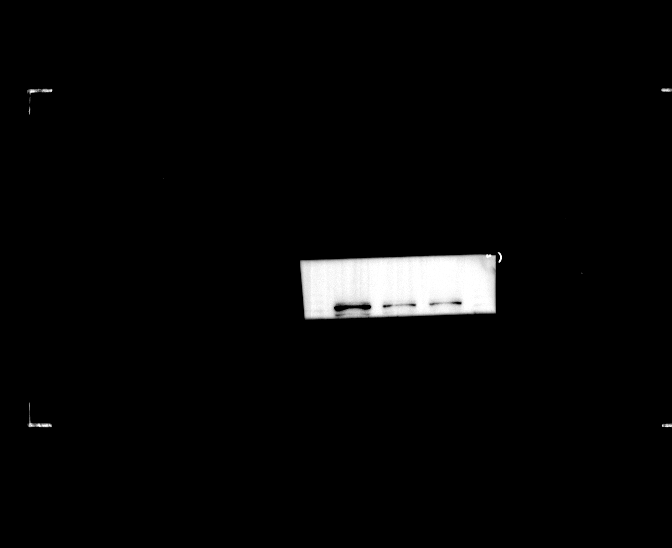


TRIM36


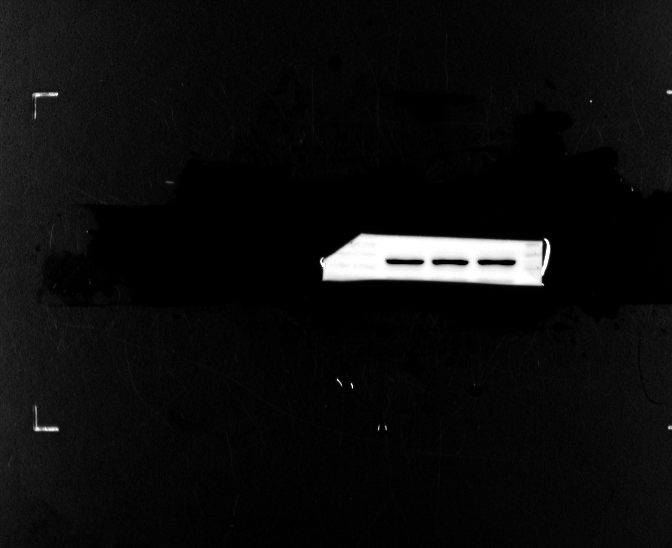


a-Tublin

Figure 9F.


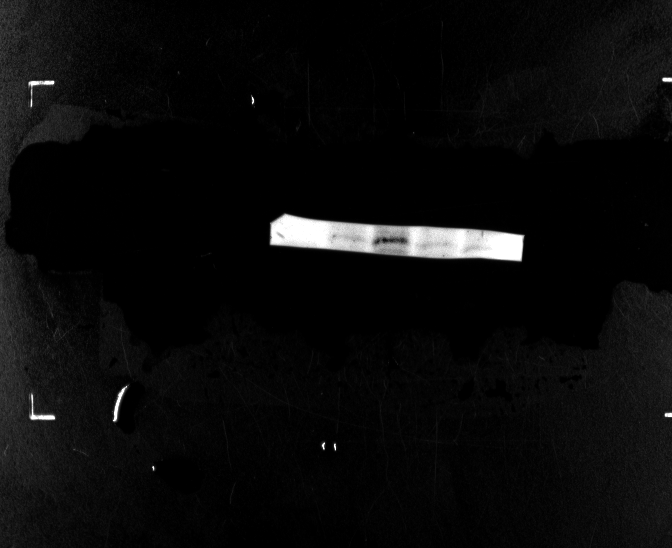


PAGE1


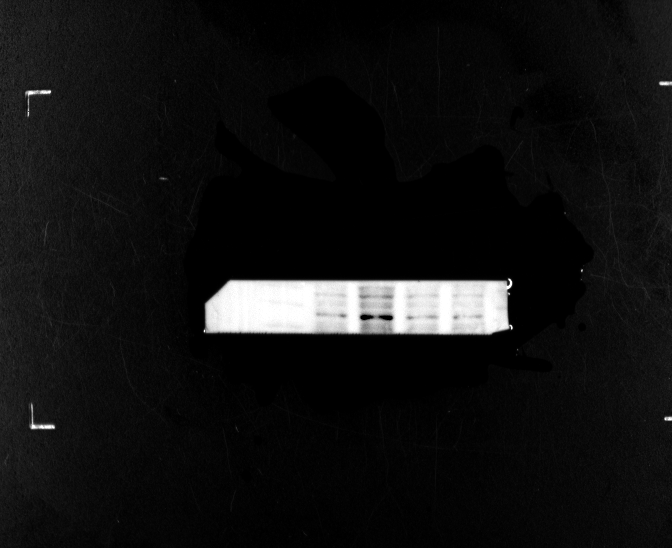


CDK5R2


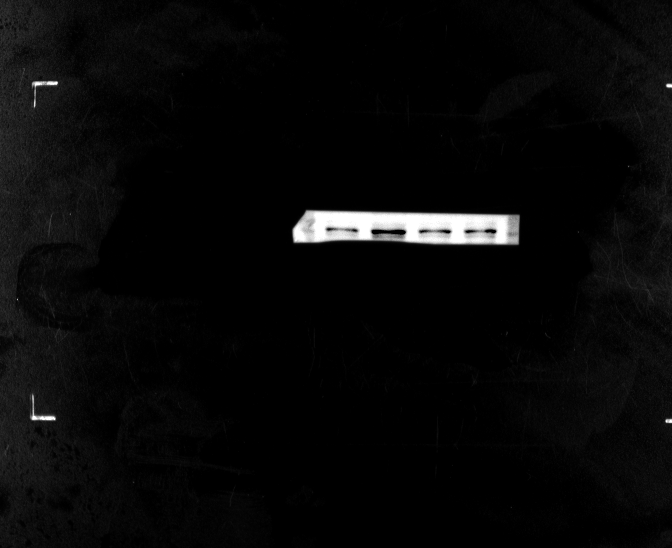


CYP26B1


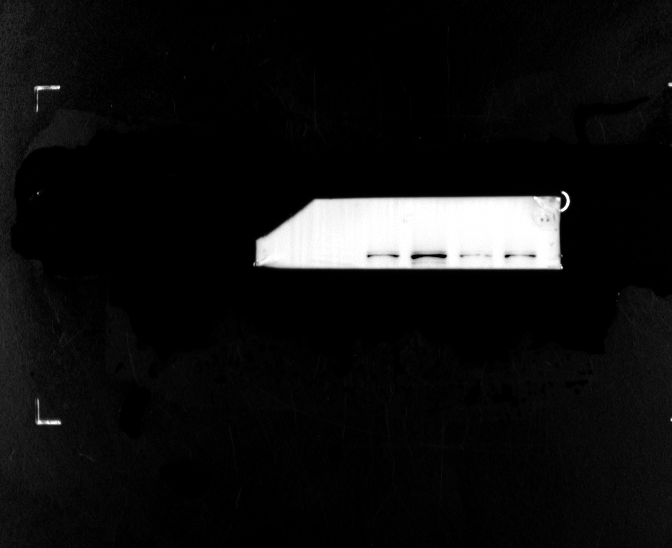


TRIM36


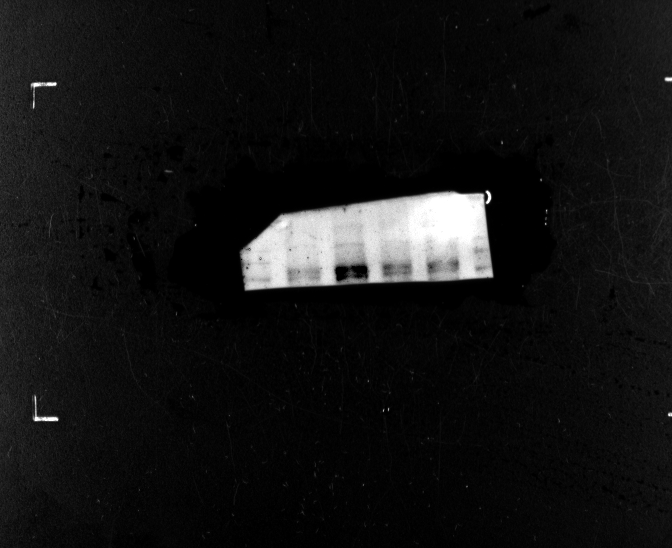


DCAF8L1


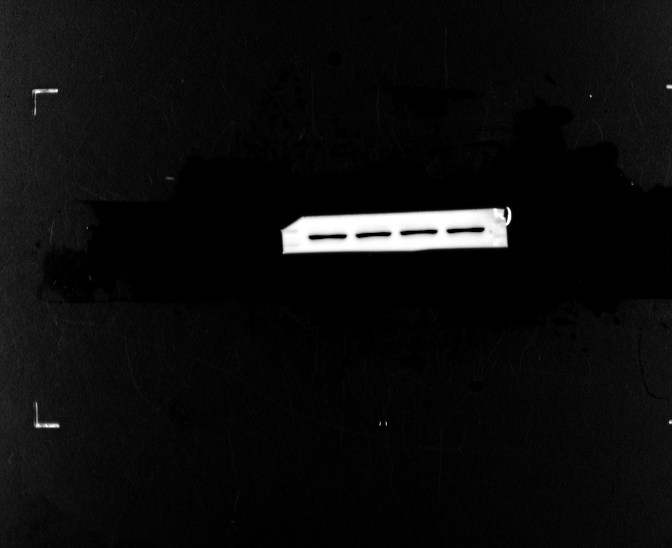


a-Tublin

DCAF8L1
